# Effects of future climate and land use changes on runoff in tropical regions of China

**DOI:** 10.1038/s41598-024-81754-8

**Published:** 2024-12-28

**Authors:** Shiyu Xue, Xiaohui Guo, Yanhu He, Hao Cai, Jun Li, Lirong Zhu, Changqing Ye

**Affiliations:** 1 School of Ecology, Hainan University, Haikou, 570228 China; 2 Institute of Environmental and Ecological Engineering, Guangdong University of Technology, Guangzhou, 510006 China; 3 School of Tourism, Hainan University, Haikou, 570228 China; 4 Key Laboratory of Agro-Forestry Environmental Processes and Ecological Regulation of Hainan Province, Haikou, 570228 China

**Keywords:** Climate change, Land use, Hydrological simulation, SWAT model, Hainan Island, Ecology, Hydrology

## Abstract

Climate change and human activities are the primary drivers influencing changes in runoff dynamics. However, current understanding of future hydrological processes under scenarios of gradual climate change and escalating human activities remains uncertain, particularly in tropical regions affected by deforestation. Based on this, we employed the SWAT model coupled with the near future (2021–2040) and middle future (2041–2060) global climate models (GCMs) under four shared socioeconomic pathways (SSP1-2.6 (SSP1 + RCP2.6), SSP2-4.5 (SSP2 + RCP4.5), SSP3-7.0 (SSP3 + RCP7.0), and SSP5-8.5 (SSP5 + RCP8.5)) from the CMIP6 and the CA-Markov model to evaluate the runoff response to future environmental changes in the Dingan River Basin (DRB). The quantification of the impacts of climate change and land use change on future runoff changes was conducted. The results revealed a non-significant increasing trend in precipitation during the historical period (1999–2018). Furthermore, all three future scenarios (SSP1-2.6, SSP3-7.0, and SSP5-8.5) exhibited an upward trend in precipitation from 2021 to 2060. Notably, the SSP5-8.5 scenario demonstrated a highly significant increase (*P* < 0.01), while the SSP2-4.5 scenario displayed a non-significant decreasing trend. The future precipitation pattern exhibits a decrease during spring and winter, while showing an increase in summer and autumn. The temperature exhibited a significant increase (*P* < 0.05) across the four future scenarios, with amplitudes of 0.24 °C/(10 years), 0.36 °C/(10 years), 0.36 °C/(10 years), and 0.50 °C/(10 years) for SSP1-2.6, SSP2-4.5, SSP3-7.0, and SSP5-8.5 respectively. The future trend of land use change entails a continuous increase in cultivated land and a corresponding decrease in artificial forest land. By 2032, the area of cultivated land is projected to witness a growth of 4.10%, while artificial forest coverage will experience a decline of 4.45%. Furthermore, by 2046, the extent of cultivated land is anticipated to expand by 4.41%, accompanied by a reduction in artificial forest cover amounting to 5.39%. The average annual runoff during the historical period was 53.35 m³/s, and the Mann-Kendall (MK) trend test showed that it exhibited a non-significant increasing trend. Compared with the historical period, the comprehensive impact of climate change and land use will cause changes in the runoff by 0.49%, 1.98%, − 3.13%, and 3.65% for the scenarios of SSP1-2.6, SSP2-4.5, SSP3-7.0, and SSP5-8.5 in the near future, and − 3.24%, 1.30%, − 3.75% and 18.24% in the middle future respectively. The intra-annual variations in future runoff suggest an earlier peak and a more concentrated distribution of runoff during the wet season (May to October). Compared to historical periods, the total runoff in the wet season under SSP1-2.6, SSP2-4.5, SSP3-7.0, and SSP5-8.5 scenarios increased by 6.53%, 8.91%, 7.17%, and 7.39%, respectively. The research findings offer significant insights into the future hydrological processes in tropical regions, while also serving as a valuable reference for watershed water resource management and disaster control.

**Supplementary Information:**

The online version contains supplementary material available at 10.1038/s41598-024-81754-8.

## Introduction

Climate change and land use change are the main drivers of runoff change^1–4^. Runoff changes are intricately linked to human life, influencing multiple dimensions of daily activities, agricultural production, and industrial development. As of 2022, the global population has reached 8 billion, and the ongoing population growth is intensifying climate change and alterations in land use^5^. Human activities have intensified the emission of greenhouse gases, thereby further accelerating the trend of global climate change. For example, global warming may lead to an increase in precipitation and enhanced atmospheric evaporation, consequently increasing and decreasing runoff, respectively^3,6,7^. Precipitation directly influences the volume and distribution patterns of runoff, serving as a significant source of runoff^8,9^. Climate change has also altered vegetation’s water utilization strategies. Increasing temperatures disrupt the growth cycle of plants, leading to an acceleration in respiration and transpiration processes, thereby modifying their capacity for water absorption^10–12^. The increase in CO_2_ concentration can induce atmospheric warming through direct radiative forcing, potentially resulting in increased precipitation and enhanced atmospheric evaporation, thereby augmenting and diminishing runoff, respectively^7^. Additionally, the elevation of CO_2_ may lead to a reduction in stomatal conductance and transpiration rates of plants, consequently contributing to an increase in runoff^13–15^. Singh, et al.^9^ study indicates that the average annual runoff of the Sutlej River, India, is projected to increase by 0.79–1.43% under the SSP585 scenario during the period from 2050 to 2080, whereas an increase of 0.87–1.10% is anticipated under the SSP245 scenario. Shrestha et al.^16^ research conducted in the Songkhram River basin, Thailand, indicates that climate change is projected to lead to a reduction in streamflow of 19.5% and 24% under the RCP4.5 and RCP8.5 scenarios, respectively, for the period from 2010 to 2099. This suggests that there is a high level of uncertainty regarding the impact of future climate change on runoff, and significant differences exist in the region’s hydrological responses; therefore, it is essential to conduct comprehensive research on the regional hydrological response to future climate change.

Human activities are progressively emerging as the primary driving force influencing land use. The exponential growth in population has resulted in an escalating demand for agricultural land. The research findings indicate that agricultural irrigation water accounts for approximately 70% of the global freshwater resources^17–19^. The construction of reservoirs and dams, as well as the rapid increase in domestic water consumption resulting from urbanization, have directly influenced surface runoff^20^. Yu et al.^21^ research conducted in the northwest region of China indicates that the “Grain for Green” accounts for 49.6% of the observed reduction in runoff. A study conducted in Uruguay indicates that when the area of afforested land reaches approximately 15% of the total watershed area, there is a significant reduction in runoff^22^. Studies have shown that land use change contributes to an increase in global runoff by 73–81% ^7^. This unequivocally demonstrates that the influence of land use change on runoff is significant and should not be overlooked.

The hydrological response to environmental changes has been extensively investigated by scholars through rigorous research^23–30^. However, some studies exhibit limitations in the selection and processing of climate and land use data, resulting in a comparatively higher degree of uncertainty in the research findings. For example, by simply increasing or decreasing temperature and precipitation, simulate future climate change^1,31,32^. Utilizing extreme land use conversion scenarios to examine the effects of future land use change on runoff, such as complete transformation of all cropland into grassland and all wasteland into forest^2,33^. The aforementioned methods provide a structured approach for quantifying the impacts of future climate and land use changes on runoff; however, considering the inherent complexities and variability of climate, along with the substantial disturbances induced by human activities, this research methodology is clearly inadequate. Moreover, research that integrates future climate change and land use change to forecast future runoff variations remains relatively limited, particularly in regions experiencing tropical deforestation.

Ding’an River serves as a vital water source in Hainan Province, playing a critical role in regional ecological balance and economic development. In recent years, the river basin has experienced frequent conversions from natural forests to rubber plantations, resulting in a noticeable decline in runoff^34^. Accurate prediction of the effects of future climate change and land use alterations on runoff is essential for mitigating the adverse impacts associated with climate variability, including droughts, floods, and the degradation of ecosystem goods and services. Based on this, we employed the SWAT model in conjunction with GCMs and the CA-Markov model to simulate the response patterns of runoff in the DRB to future climate change and land use changes from 2021 to 2060. The impacts of future climate change and land use change on runoff were analyzed both independently and in conjunction. The research findings offer empirical support and a reference framework for water resources planning, management, and sustainable development in the background of future environmental changes.

## Materials and methods

### Study area and relevant data

#### Study area

**Fig. 1:**
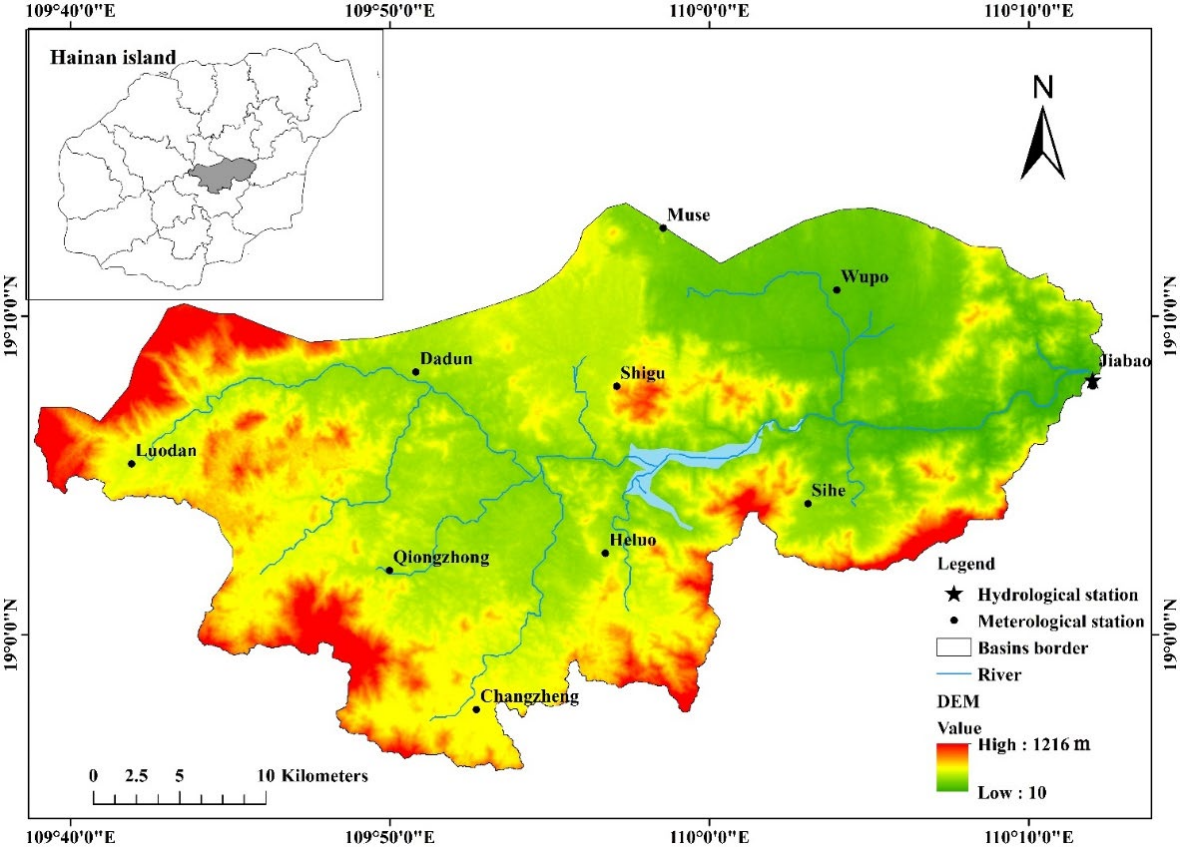
Diagram of Dingan River Basin and hydrometeorological monitoring stations. The maps were created by the authors using ArcGIS 10.2 (Environmental Systems Research Institute, USA; https://www.esri.com/).

The Dingan River Basin (DRB) serves as the northern tributary of the Wanquan River, originating from Fengmenling in Qiongzhong county (Fig. 1). It spans a total length of 88 km and encompasses a drainage area measuring 1149 km^2^. The DRB is situated within the tropical monsoon region, experiencing an annual precipitation of 2453 mm. The distinct wet season (May–October) and dry season (November–April) are evident.

#### Database

The precipitation, wind speed, maximum and minimum temperatures, light duration, and relative humidity daily data spanning from 1960 to 2018 were sourced from the Hainan Provincial Meteorological Bureau and the China Meteorological Service Center. The daily runoff data of the Jiabao hydrological station spanning from 1967 to 2016 was acquired from the Water Affairs Department of Hainan Province.

The daily precipitation, minimum temperature, and maximum temperature global climate scenario data (historical, SSP1-2.6, SSP2-4.5, SSP3-7.0, and SSP5-5.8) of 1960–2060 are from the EC-Earth3-Veg in the CMIP6 models provided by the World Climate Research Program (WCRP) official website (https://esgf-node.llnl.gov/search/cmip6). We selected EC-Earth3-Veg for this study primarily because multi-climate ensemble means generally demonstrate limited effectiveness in capturing the amplitude of observed multi-decadal precipitation variability^35–38^. Furthermore, EC-Earth3-Veg has undergone extensive validation through numerous studies, confirming its high accuracy in simulating future climate scenarios^39–43^.

**Fig. 2:**
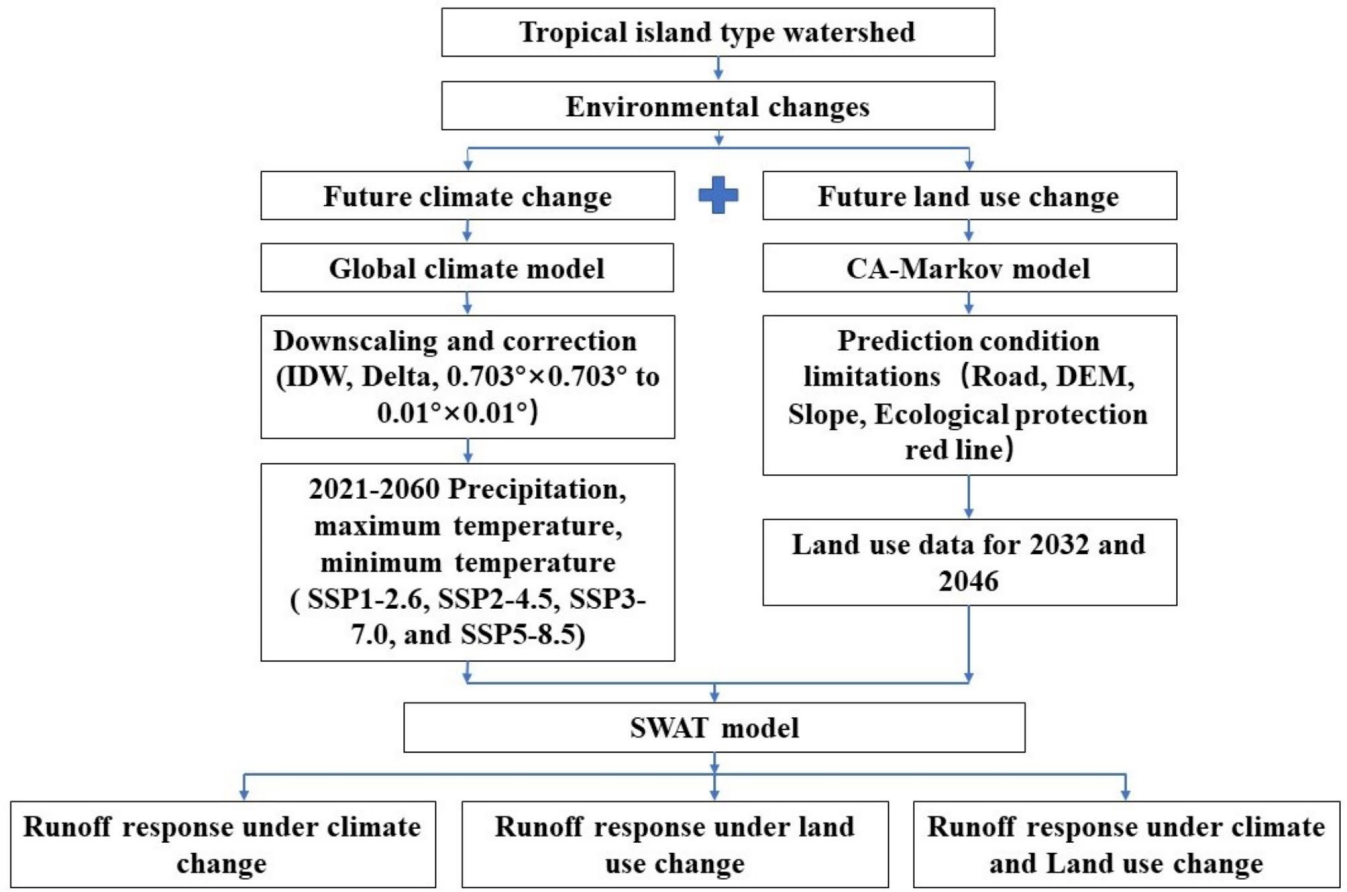
Schematic representation of the research framework.

**Fig. 3:**
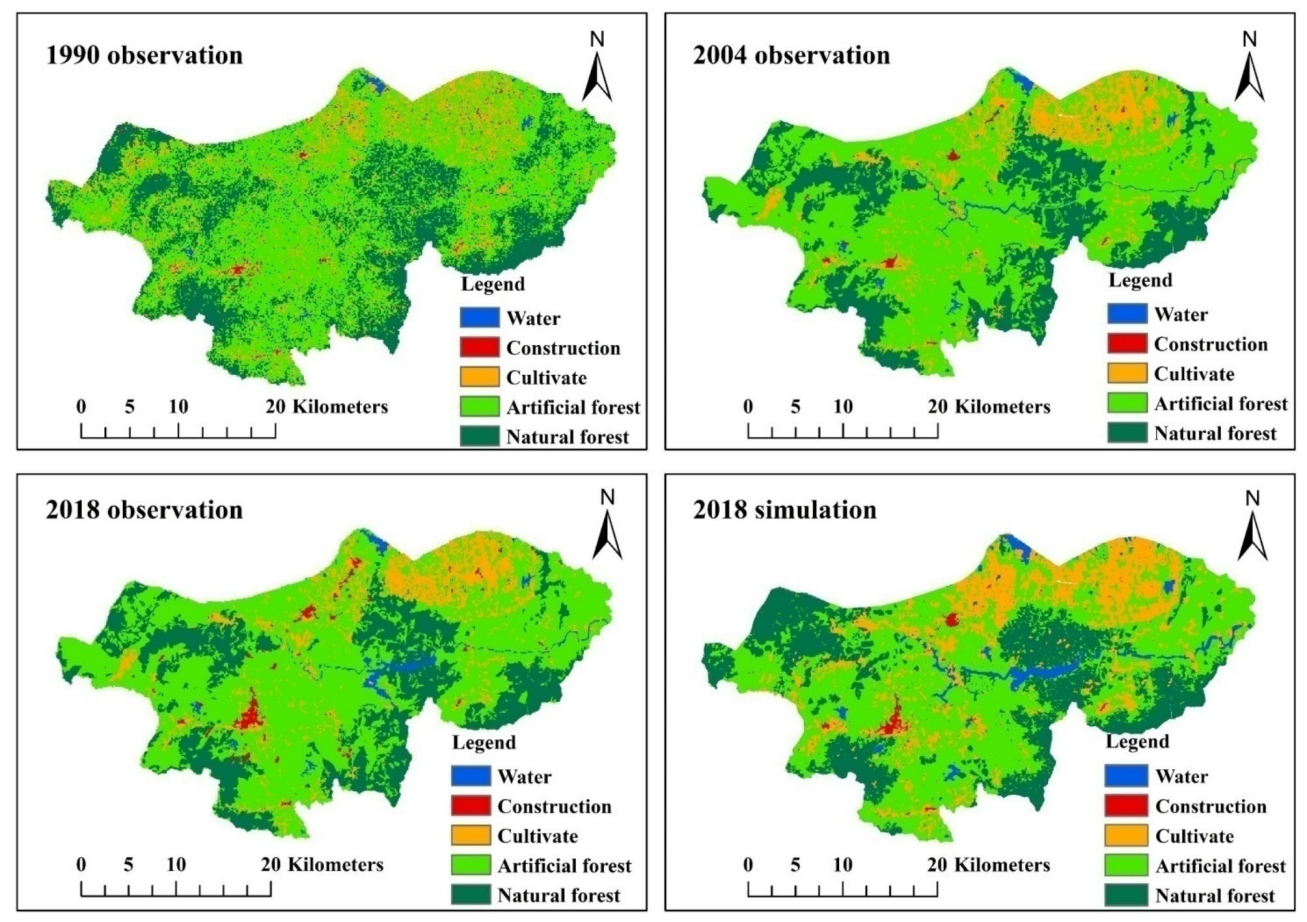
Observation and simulation results of land use dynamics in the Dingan River Basin for the years 1990, 2004, and 2018. The maps were created by the authors using ArcGIS 10.2 (Environmental Systems Research Institute, USA; https://www.esri.com/).

The primary research framework of this study is illustrated in Fig. 2. The land use data of 1990, 2004, and 2018 were acquired by interpreting remote sensing images from the Geospatial Data Cloud Website (https://www.gscloud.cn/#page1/3) using ENVI software at a spatial resolution of 30 m × 30 m (Fig. 3). The road data within the watershed comes from the OpenStreetMap platform (https://www.Openstreetmap.org). The boundary data of the ecological protection red line is sourced from the Hainan Provincial People’s Government, while the slope data is derived through analysis of Digital Elevation Model (DEM) data using ArcGIS software. The DEM data with a spatial resolution of 30 m × 30 m was obtained from the official website of the Geospatial Data Cloud (https://www.gscloud.cn/). Table 1 presents a comprehensive overview of the data necessary for this study along with their corresponding source information.

**Table 1:** Description of data and sources for research applications in the Dingan River Basin.

Data type	Scale	Data description	Source
Digital Elevation Model	1:200,000	Elevation, overland and channel slopes and lengths	Geospatial Data Cloud
Land use	1:100,000	Land use classifications, 1990, 2004, 2018	Geospatial data cloud
Soil properties	1:1000,000	Soil physical and chemical properties	Harmonized World Soil Database
Climate data	daily	Precipitation, wind speed, daily maximum and minimum air temperature, relative humidity, and solar radiation, 1960–2018	Hainan Meteorological Bureau, China Meteorological Data Service Centre
Runoff data	daily	Cross section runoff of Jiabao, 1967–2016	Hainan Water Resources Department
Future climate data	daily	historical, SSP1-2.6, SSP2-4.5, SSP3-7.0, and SSP5-5.8, 1960–2060	EC-Earth3-Veg of CMIP6 in WCRP
Road data	1:100,000	National, Provincial, County, and Township roads	OpenStreetMap platform
Ecological protection red line	1:100,000	Protected area boundaries	Hainan Provincial People’s Government
Slope data	1:200,000	Slope of the terrain	ArcGIS processes DEM

### Prediction of future climate change

#### Downscaling and correction of climate model data

The resolution of GCMs data is too coarse when directly applied to the Hydrological model for watershed-scale research. Therefore, in this study, the statistical downscaling method known as Inverse Distance Weight (IDW) interpolation is employed for spatially downsizing climate scenario data. Subsequently, the Delta method is utilized for data correction.

The mathematical formulation of IDW is as follows:

1 $$\:\begin{array}{c}{\widehat{Z}}_{0}=\sum_{i=0}^{n}\left({Z}_{i}{Q}_{i}\right)\end{array}$$

where $\:{\widehat{Z}}_{0}$ is the estimated value at point (x_0_, y_0_), *Z*_*i*_ is the observation value at point (x_i_, y_i_), *Q*_*i*_ is the weight coefficient corresponding to the interpolation point and the observation point, and *n* is the number of interpolation points.

2 $$\:\begin{array}{c}{Q}_{i}=\frac{f\left({d}_{ej}\right)}{\sum_{j=1}^{n}f\left({d}_{ej}\right)}\end{array}$$

where *Q*_*i*_ is the weight coefficient, *d*_*ej*_ is the distance between observation points and interpolation points, *n* is the number of observation points.

3 $$\:\begin{array}{c}f\left({d}_{ej}\right)=\frac{1}{{d}_{ej}^{b}}\end{array}$$

where, *b* is an appropriate constant. When the *b* value is 1 or 2, it is inverse distance reciprocal interpolation and inverse distance reciprocal square interpolation.

The Delta method is simple, can simulate multiple stations at the same time, and can better simulate the changes of temperature and precipitation, and is widely used. The specific formula is as follows:

4 $$\:\begin{array}{c}{T}_{f}={T}_{o}+\left({T}_{Gf}-{T}_{Go}\right)\end{array}$$

where, *T*_*f*_ is the corrected air temperature, °C; *T*_*o*_ is the historical measured temperature, °C; *T*_*Gf*_ is the future temperature predicted by the climate model, and *T*_*Go*_ is the historical temperature simulated by the climate model.

5 $$\:\begin{array}{c}{P}_{f}={P}_{0}\frac{{P}_{Gf}}{{P}_{Go}}\end{array}$$

where *P*_*f*_ is the corrected precipitation, mm; *P*_*0*_ is the historical measured precipitation, mm; *P*_*Gf*_ is the future precipitation predicted by the climate model, and *P*_*Go*_ is the historical precipitation simulated by the climate model.

#### Validation of climate simulation results

The correlation coefficient (*R*^2^) and the Nash-Sutcliffe efficiency (*E*_*NS*_) coefficient are used to validate the availability of model simulation results.

6 $$\:\begin{array}{c}{R}^{2}={\left[\frac{\underset{i=1}{\sum\:^{n}}\left({O}_{i}-\stackrel{-}{O}\right)\left({P}_{i}-\stackrel{-}{P}\right)}{\sqrt{\underset{i=1}{\sum\:^{n}}{\left({O}_{i}-\stackrel{-}{O}\right)}^{2}\underset{i=1}{\sum\:^{n}}{\left({P}_{i}-\stackrel{-}{P}\right)}^{2}}}\right]}^{2}\end{array}$$

where *O*_*i*_ is the observed data, $\stackrel{-}{O}$ is the mean value of observed data, *P*_*i*_ is the simulated data, and $\stackrel{-}{p}$ is the mean value of simulated data.

7 $$\begin{array}{*{20}c} {E_{{NS}} = 1 - \frac{{\sum\nolimits_{i = 1}^{n} {\left( {Q_{{sim,i}} - Q_{{mea,i}} } \right)^{2} } }}{{\sum\nolimits_{i = 1}^{n} {\left( {Q_{{mea,i}} - \bar{Q}_{{mea}} } \right)^{2} } }}} \\ \end{array}$$

where *Q*_*sim, i*_ is the simulated value, *Q*_*mea, i*_ is the observed value, *Q*_*mea*_ is the mean observed data, and *n* is the total number of observations.

### Prediction of future land use

#### CA-Markov model

The CA-Markov model is a coupling model of the set Cellular Automata model and the Markov model. The model integrates the simulation of complex spatial dynamics and a long-term prediction capability module, making it widely applicable for simulating and predicting long-term changes in land use structure^44–46^.

#### Simulation steps

The simulation and prediction of land use are conducted using IDRISI 17.2 software, following the specific steps outlined below.

1. Data preparation. The land use data (Fig. 3), DEM data, and slope data in the study area should be uniformly converted into ASCII format that is compatible with IDRISI software.
2. The construction of the Markov Transfer Matrix. In IDRISI software, land use data is classified into five categories: Water, Built, Farmland, Artificial forest, and Natural forest. The Markov module in the software is executed to calculate the area and probability transfer matrix for two times intervals (1990–2004 and 2004–2018), with a land use data interval of 14 years. Additionally, a proportion error of 0.15 is set.
3. Creation of suitability atlas. The “decision wizard” module of IDRISI software is utilized to generate the suitability atlas, incorporating road distribution data, ecological protection red line data, DEM, and slope data in the basin as constraint conditions for land use prediction to enhance the realism and rationality of prediction outcomes. Ultimately, the land-use suitability atlas of the study area is constructed using the “Collection Editor” module.
4. Land use simulation. The CA-Markov model in IDRISI software was utilized for land use prediction, incorporating the actual land use situation in 2004, the transfer matrix from 1990 to 2004, and the constructed suitability atlas as constraints. A selection of 5 × 5 filters was applied with automatic iteration performed 14 times to simulate the land use status of DRB in 2018. By utilizing the actual land use data from 2018 as a reference for simulation, and incorporating the land use transfer matrix from 2004 to 2018, and suitability atlas constructed as constraints, a 5 × 5 filter was selected with automatic iteration performed 14 times to simulate the future land use status in the DRB region by 2032. Similarly, employing the aforementioned settings, after conducting 28 iterations, we can obtain the land use of the DRB by 2046.

#### Validation of land use simulation results

The effectiveness of the CA-Markov model simulation is assessed through the Kappa index, which is formulated as follows:

8 $$\:\begin{array}{c}k=\frac{{p}_{0}-{p}_{c}}{1-{p}_{c}}\end{array}$$

where *P*_*0*_ is the probability value of comparison between actual land use and simulated land use; *P*_*c*_ is the expected value of the correct ratio of simulated land use. The Kappa value ranges from 0 to 1, with higher values indicating a more favorable simulation effect.

### SWAT model construction

The Soil and Water Assessment Tool (SWAT) model is a widely utilized semi-distributed hydrological and water quality model, which operates on the fundamental principle of water balance^47–49^.

According to previous research on the DRB, the runoff underwent a significant transformation in 1990 as a result of both climate change and human activities^34^. In order to avoid the influence of environmental changes on the uncertainty of model parameters, the calibration and validation of the SWAT model were carried out using data before the runoff abrupt change point. Therefore, the actual measured monthly scale runoff data from Jiabao station from 1967 to 1968 was used as the warm-up period of the SWAT model, 1969–1979 as the calibration period, and 1980–1989 as the validation period. Utilize *R*^2^ (Eq. 6) and the *E*_*NS*_ (Eq. 7) for the assessment of simulation outcomes derived from the SWAT model.

### Scenarios setting

Four scenarios were set up based on different combinations of meteorological and land use data used to drive the SWAT model, as shown in Table 2. In this study, we employ the land use of 2032 to represent the land use of the near future (2021–2040), and the land use of 2046 to represent the land use of the middle future (2041–2060).

**Table 2:** Scenario setting for SWAT model-driven dataset.

Scenario	Climate scenario	Land use scenario
S_0_	1999–2018	2018
S_1_	2021–2040, 2041–2060	2018
S_2_	1999–2018	2032, 2046
S_3_	2021–2040, 2041–2060	2032, 2046

## Results

### Calibration and validation of model simulation results

#### Validation of downscaled data from GCMs

The *R*^2^ and *E*_*NS*_ metrics are employed to evaluate the suitability of downscaling climate data by comparing daily precipitation and temperature measurements from the DRB dataset (1960–2014) with downscaled daily data derived from the EC-Earth3-Veg Climate model. The closer the *R*^2^ and *E*_*NS*_ values approach 1, the higher the fidelity of model simulation. The validation results demonstrate that the *R*^2^ and *E*_*NS*_ values for precipitation are 0.516 and 0.521, respectively. For maximum temperature, the *R*^2^ and *E*_*NS*_ values are 0.778 and 0.783, respectively; while for minimum temperature, the *R*^2^ and *E*_*NS*_ values are 0.856 and 0.857, respectively (Table 3). Several factors, including complex topography and the influence of monsoons, may impact the accuracy of precipitation forecasts^50^. Fortunately, validation results indicate that the downscaled climate data yield satisfactory performance^51,52^.

**Table 3:** Validation of downscaling results for GCMs.

Classify	Indicator	
	*R* ^2^	E_NS_
Precipitation	0.516	0.521
Maximum temperature	0.778	0.783
Minimum temperature	0.856	0.857

#### Validation of land use prediction results

We compared the actual land use data for 2018 with the CA-Markov simulated land use data for 2018 (Fig. 2). The comparative analysis results show that the Kappa coefficient between the actual values and the simulated values is 0.776, indicating that the simulation accuracy is high and meets the model simulation accuracy requirements.

#### Calibration and validation of SWAT model

The Soil and Water Assessment Tool Calibration Uncertainty Program (SWAT-CUP) is utilized for the calibration and validation of the SWAT model, with the Nash-Sutcliffe efficiency (*E*_*NS*_) as the objective function^51,53,54^. The SWAT-CUP program has been specifically developed to enhance the calibration efficiency of the SWAT model^47,48,55^. The Sequential Uncertainty Fitting version 2 (SUFI-2) program is chosen for specific operations due to its relatively straightforward implementation, efficient running times, and commendable simulation performance. The optimal parameter combination for the SWAT model was obtained through repeatedly iterated by SWAT-CUP. The sensitivity analysis results of the runoff parameters in the DRB are shown in Table 4. The monthly runoff data from the Jiabao Station from 1969 to 1979 was used as the calibration period for the model, while the monthly runoff data from 1980 to 1989 was used as the validation period. The final calibration results show *R*^2^ is 0.85, *E*_*NS*_ is 0.84, while the validation results indicate *R*^2^ is 0.86, *E*_*NS*_ is 0.83, demonstrating excellent model simulation performance that meets the required accuracy for this study.

**Table 4:** Sensitivity analysis of runoff parameters in the DRB.

Ranking of Parametric Sensitivity	Designation of Parameter	t-stat	*P*-Value	Min value	Max value	Fitted value
1	R_CN2.mgt	32.61	0.00	− 0.20	0.20	− 0.13
2	R_SOL_K.sol	− 15.42	0.00	− 0.80	0.80	− 0.78
3	V_ALPHA_BF.gw	8.13	0.00	0.00	1.00	0.23
4	V_GWQMN.gw	− 4.00	0.00	0.00	5000.00	1665.00
5	V_CH_N2.rte	− 2.05	0.04	− 0.01	0.30	0.15
6	R_SOL_AWC.sol	− 1.88	0.06	− 0.20	0.40	− 0.19
7	V_GW_REVAP.gw	1.55	0.12	0.02	0.20	0.16
8	V_RCHRG_DP.gw	− 1.45	0.15	0.00	1.00	0.30
9	V_CANMX.hru	− 0.89	0.38	0.00	100.00	53.50
10	V_GW_DELAY.gw	0.88	0.38	0.00	500.00	367.40
11	V_CH_K2.rte	− 0.41	0.68	− 0.01	500.00	83.63
12	V_ESCO.hru	0.29	0.77	0.00	1.00	0.47
13	V_REVAPMN.gw	0.04	0.97	0.00	500.00	0.01

V− means the parameter value is to be replaced by the given value; R-means the parameter value is multiplied by (1 + a given value).

### Response of runoff to future climate change

The statistical analysis of climate series, derived from the downscaling of the EC-Earth3-Veg climate model of various scenarios is conducted. The Mann Kendall (MK) trend analysis reveals an increasing trend in historical precipitation patterns. However, under the SSP2-4.5 scenario, the future (2021–2060) shows a statistically insignificant decrease in precipitation trends. Conversely, the scenarios of SSP1-2.6, SSP3-7.0, and SSP5-8.5 exhibit an upward trend in precipitation changes. Notably, SSP5-8.5 demonstrates a significant increase trend (*P* < 0.01) (Fig. S1).

**Fig. 4:**
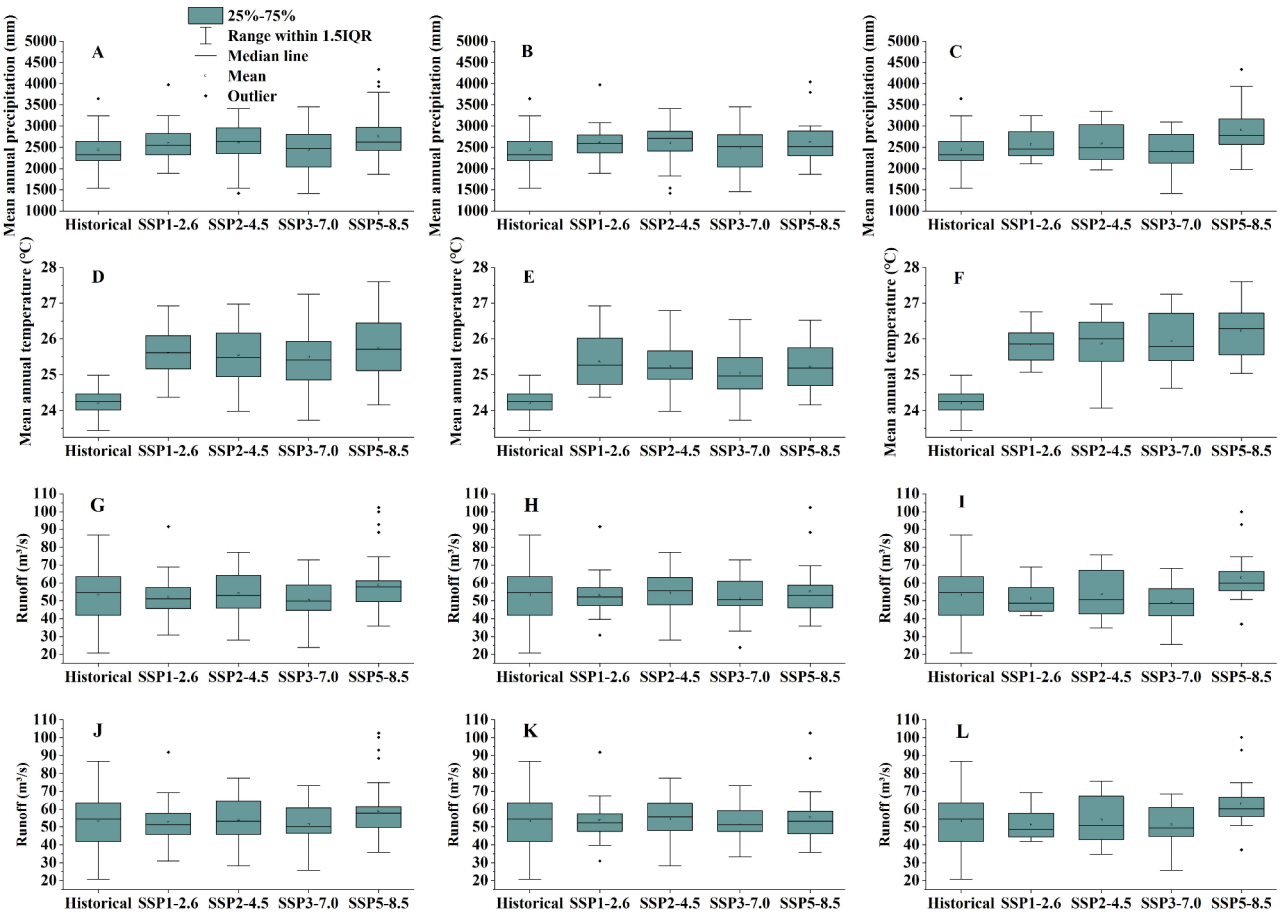
Box plots depicting precipitation, temperature, and runoff across various climate models during different time periods. (**A**: Precipitation for 2021–2060;** B**: Precipitation for 2021–2040;** C**: Precipitation for 2041–2060;** D**: Temperature for 2021–2060;** E**: Temperature for 2021–2040;** F**: Temperature for 2041–2060;** G**: Runoff under climate impact for 2021–2060;** H**: Runoff under climate impact for 2021–2040;** I**: Runoff under climate impact for 2041–2060;** J**: Combined impact of climate change and land use change on runoff for 2021–2060;** K**: Combined impact of climate change and land use change on runoff for 2021–2040;** L**: Combined impact of climate change and land use change on runoff for 2041–2060).

The findings indicate a decrease in precipitation during the middle future (2041–2060) under the SSP3-7.0 scenario compared to the historical period, while an increase in precipitation is projected for the future periods (2021–2060) under the SSP1-2.6, SSP2-4.5, and SSP5-8.5 scenarios. The annual average precipitation under the SSP1-2.6, SSP2-4.5, SSP3-7.0, and SSP5-8.5 scenarios increased by 141.59 mm, 157.43 mm, 9.84 mm, and 320.78 mm respectively compared to the historical period in the years 2021–2060 (Fig. 4A). The annual precipitation amounts will show SSP5-8.5 > SSP2-4.5 > SSP1-2.6 > SSP3-7.0 in different periods in the future (Table 5; Fig. 4A–C). The test results indicate a progressive widening trend in the disparities among various climate scenarios over time (Fig. 4B and C).

**Table 5:** Variations in annual precipitation under diverse climate scenarios across different temporal periods (mm).

Time	Scenario				
	Historical	SSP1-2.6	SSP2-4.5	SSP3-7.0	SSP5-8.5
1999–2018	2446.77	–	–	–	–
2021–2040	–	2603.40	2611.60	2511.32	2619.57
2041–2060	–	2573.33	2596.80	2414.06	2915.53
2021–2060	–	2588.36	2604.20	2456.61	2767.55

**Fig. 5:**
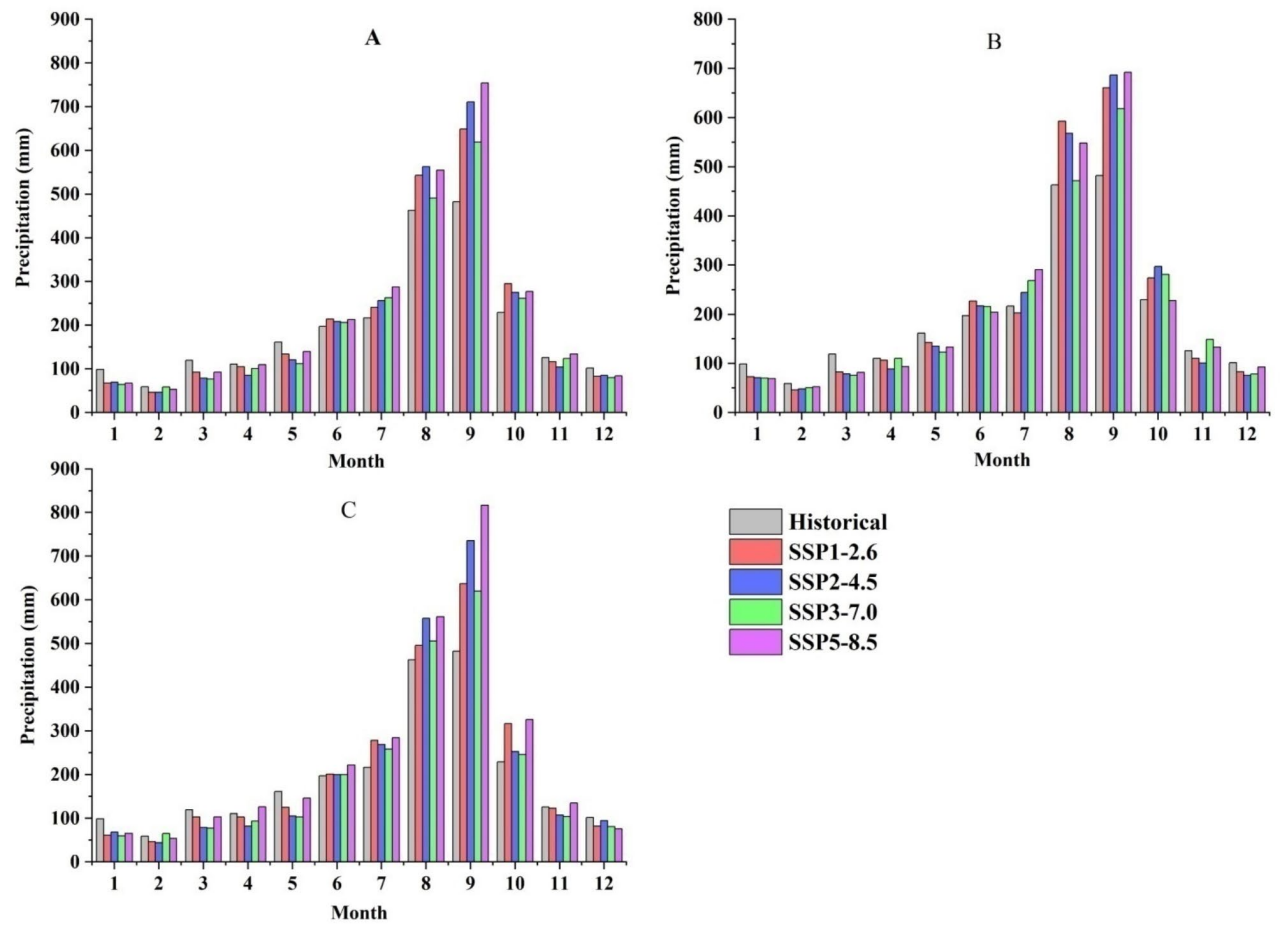
Distribution pattern of intra-annual precipitation under different climate scenarios in different periods (**A**: 2021–2060;** B**: 2021–2040;** C**: 2041–2060).

The intra-annual precipitation in the four future climate scenarios demonstrates a tendency towards increased precipitation during wet season (May–October) and decreased precipitation during dry season (November–April), when compared to historical periods (Fig. 5). The annual precipitation in the historical period is characterized by a distribution of 73.98% during the wet season and 26.02% during the dry season. The wet season precipitation of SSP1-2.6, SSP2-4.5, SSP3-7.0, and SSP5-8.5 accounts for 80.27%, 81.97%, 79.47%, and 80.44% of the annual precipitation respectively, while the dry season contributes to 19.73%, 18.03%, 20.53%, and 19.56% respectively. Compared to historical periods, future winter and spring precipitation will decrease, while summer and autumn precipitation will increase.

The MK trend analysis of annual average temperature under the four future climate scenarios from 2021 to 2060 reveals a statistically significant increasing trend (*P* < 0.05). Specifically, the temperature exhibits an upward trend in SSP1-2.6, SSP2-4.5, SSP3-7.0, and SSP5-8.5 with rates of 0.24 °C/(10 years), 0.36 °C/(10 years), 0.36 °C/(10 years), and 0.50 °C/(10 years) respectively (Fig. S2). Compared to the historical average temperature of 24.22 °C, the projected average temperature increase is estimated to be 1.39 °C, 1.34 °C, 1.28 °C, and 1.51 °C under the SSP1-2.6, SSP2-4.5, SSP3-7.0, and SSP5-8.5 scenarios in the future (2021–2060) (Fig. 4D). The projected temperature increases for SSP1-2.6, SSP2-4.5, SSP3-7.0, and SSP5-8.5 in the middle future are 1.39, 1.65, 2.10, and 1.98 times higher than the corresponding near future increases (Fig. 4E,F).

Figure 3A illustrates the trend of intra-annual monthly average temperature change across different climate scenarios from 2021 to 2060. In comparison with the historical period, a significant increase in monthly average temperature is anticipated for all four future climate scenarios, and the overall pattern of change remains consistent. The trends in the monthly average temperature for both the near and middle periods of intra-annual also exhibit similar change patterns (Fig. S3BC).

The SWAT model is employed to assess the response of runoff to future changing environments by integrating different land use and climate data combination scenario datasets. By comparing the simulation results of runoff changes under scenarios S_0_ and S_1_, we can quantitatively assess the characteristics of future climate-induced alterations in runoff. The MK test results indicate an increasing trend in runoff changes under the future climate scenarios of SSP1-2.6, SSP2-4.5, SSP3-7.0, and SSP5-8.5, which the scenarios of SSP5-8.5 reaches a very significant level (*P* < 0.01) (Fig. S4).

The annual average runoff under S_1_, compared to S_0_, exhibits temporal inconsistency in both the near future (2021–2040) and the middle future (2041–2060), across the four future climate scenarios (Fig. S4; Fig. 4G–I). The runoff under the SSP1-2.6 climate scenario exhibits a marginal increase of 0.16% in the near future compared to S_0_, while experiencing a notable decrease of − 3.68% in the middle future. Under the SSP2-4.5 and SSP5-8.5 scenarios of S_1_, the runoff in the near and middle future exhibited increases of 1.67%, 0.86%, 3.31%, and 17.78% compared to S_0_, respectively (Table 6). The runoff of SSP3-7.0 exhibits a reduction in the near and middle future, with a declining ratio of − 3.71% and − 7.24% respectively, when compared to S_0_ (Table 6).

**Table 6:** Annual average runoffs under diverse climate scenarios across different time periods.

Scenario	Time					
	2021–2040			2041–2060		
	Simulation value (m^3^/s)	Change value (m^3^/s)	Rate of change (%)	Simulation value (m^3^/s)	Change value (m^3^/s)	Rate of change (%)
S_0_	53.35	–	–	53.35	–	–
S_1__SSP1-2.6	53.44	0.09	0.16	51.39	− 1.96	− 3.68
S_1__SSP2-4.5	54.24	0.89	1.67	53.81	0.46	0.86
S_1__SSP3-7.0	51.37	− 1.98	− 3.71	49.49	− 3.86	− 7.24
S_1__SSP5-8.5	55.12	1.77	3.31	62.84	9.49	17.78

### Impact of land use change on runoff

**Fig. 6:**
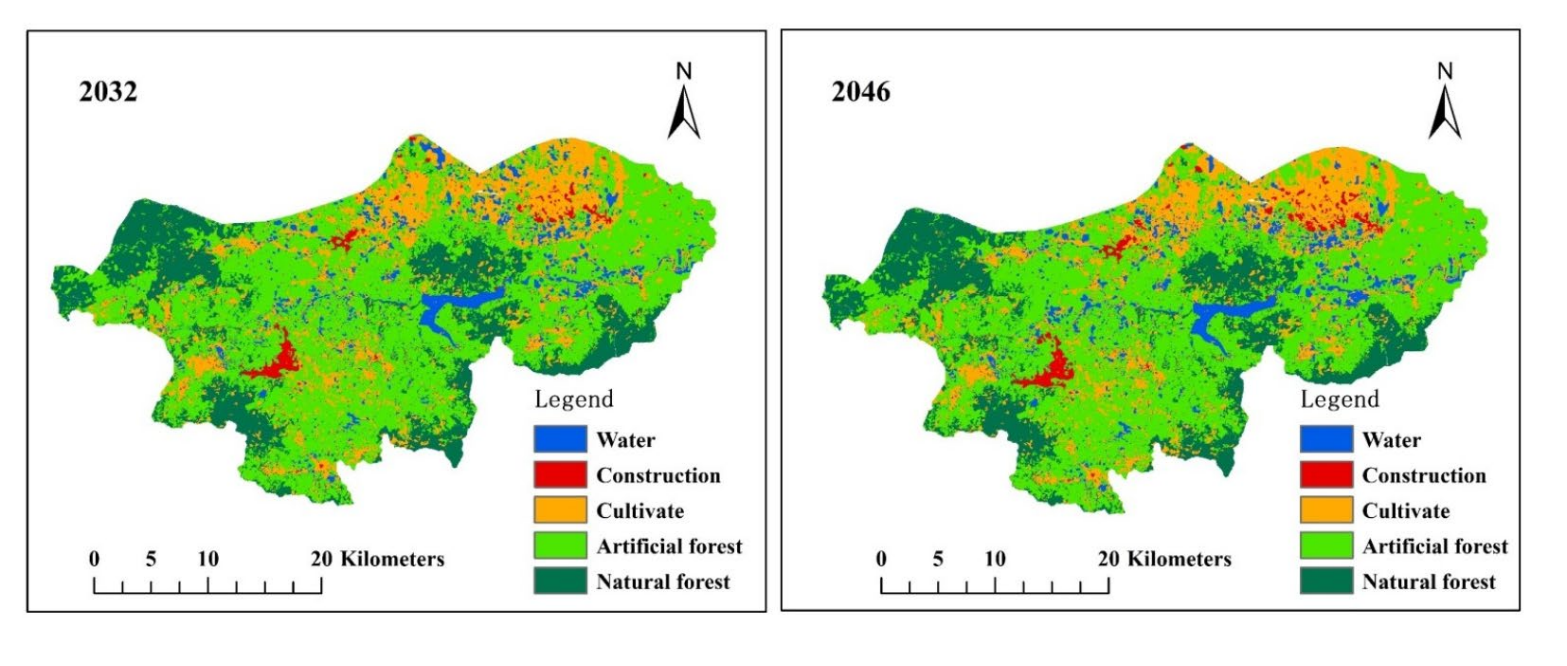
Simulation diagram depicting land use change in the Dingan River Basin for 2032 and 2046. The maps were created by the authors using ArcGIS 10.2 (Environmental Systems Research Institute, USA; https://www.esri.com/).

According to the comprehensive analysis of land use in the DRB in 2018, artificial forests, natural forests, and cultivated lands constitute the primary land uses, accounting for 57.64%, 19.92%, and 14.79% respectively. The proportions of water bodies and construction land are relatively smaller at 6.29% and 1.36% respectively. The land use change maps of the DRB for the future years 2032 and 2046 were derived using the CA-Markov model (Fig. 6). The land use in 2032 is projected to witness a marginal increase of 0.13% for water bodies, 0.34% for construction land, and a substantial growth of 4.10% for cultivated land compared to the base period of 2018. Conversely, artificial forest and natural forest are anticipated to experience reductions of − 4.45% and − 0.12%, respectively. By 2046, this changing trend will be further intensified. Compared with the base period of 2018, there will be a projected increase in the area of water bodies (0.33%), construction land (0.78%), and cultivated land (4.41%). Conversely, there is expected to be a decrease in the area of artificial forest (− 5.39%) and natural forest (− 0.13%) respectively (Table 7).

**Table 7:** Land use composition across different time periods in the Dingan River Basin (km^2^).

Land use	Time		
	Historical scenario	Future scenarios	
	2018	2032	2046
Water	72.27 (6.29%)	73.77 (6.42%)	76.06 (6.62%)
Construction land	15.63 (1.36%)	19.53 (1.70%)	24.59 (2.14%)
Cultivated land	169.94 (14.79%)	217.05 (18.89%)	220.61 (19.20%)
Artificial forest	662.28 (57.64%)	611.15 (53.19%)	600.35 (52.25%)
Natural forest	228.88 (19.92%)	227.50 (19.80%)	227.39 (19.79%)

The numbers in the parentheses in the table represent the proportion of each land use type in relation to the total watershed area.

The impact of land use change on runoff can be quantified by comparing S_2_ with S_0_ through SWAT model simulation. The findings indicate that land use change is projected to result in a 0.51% increase in annual average runoff by 2032, followed by a further rise of 0.66% by 2046. Comparing S_1_ and S_3_, the impact of future land use change on runoff can be assessed under the future four different climate scenarios. The findings reveal that, in the near future, runoff is projected to increase by 0.32%, 0.31%, 0.60%, and 0.33% respectively for SSP1-2.6, SSP2-4.5, SSP3-7.0, and SSP5-8.5 compared to the land use conditions in 2018. The runoff under the 2046 land use exhibits an increase of 0.46%, 0.43%, 3.76%, and 0.39% when compared to the corresponding values in the 2018 land use scenario for the middle future projections of SSP1-2.6, SSP2-4.5, SSP3-7.0, and SSP5-8.5 climate scenarios respectively.

### Integrated impacts of climate change and land use on runoff

#### The inter-annual variation of runoff

Implement scenario S_3_ to comprehensively analyze the integrated impact of climate and land use changes on runoff, considering variations in both climatic conditions and land use patterns. The MK change trend test reveals that the statistical Z values for the four climate scenarios (SSP1-2.6, SSP2-4.5, SSP3-7.0, and SSP5-8.5) are 0.221, 0.058, 0.384, and 3.041 respectively. This suggests that all four climate scenarios exhibit an increasing trend in future runoff changes (Fig. S5); notably, the increasing trend under the SSP5-8.5 scenario reaches a highly significant level (*P* < 0.01). In comparison to S_0_, the SSP1-2.6 scenarios under S_3_ exhibit a 0.49% increase in runoff in the near future, whereas a − 3.24% decrease in runoff is observed in the middle future. Compared with S_0_ scenario, SSP2-4.5 and SSP5-8.5 under S_3_ scenario will increase runoff by 1.98% and 3.65% in the near future, and by 1.30% and 18.24% in the middle future, respectively (Fig. 4J–L). The noteworthy point is that SSP3-7.0, under S_3_ scenario in both the near and middle future, exhibits a reduction in runoff with rates of − 3.13% and − 3.75%, respectively (Fig. 4J–L; Table 8).

**Table 8:** The combined impacts of land use and climate change on runoff.

Scenario	Land use					
	2032			2046		
	Simulation value (m^3^/s)	Change value (m^3^/s)	Rate of change (%)	Simulation value (m^3^/s)	Change value (m^3^/s)	Rate of change (%)
S_0_	53.35	–	–	53.35	–	–
S_3__SSP1-2.6	53.61	0.26	0.49	51.62	− 1.73	− 3.24
S_3__SSP2-4.5	54.41	1.06	1.98	54.04	0.69	1.30
S_3__SSP3-7.0	51.68	− 1.67	− 3.13	51.35	− 2.00	− 3.75
S_3__SSP5-8.5	55.30	1.95	3.65	63.08	9.73	18.24

#### The intra-annual variation of runoff

**Fig. 7:**
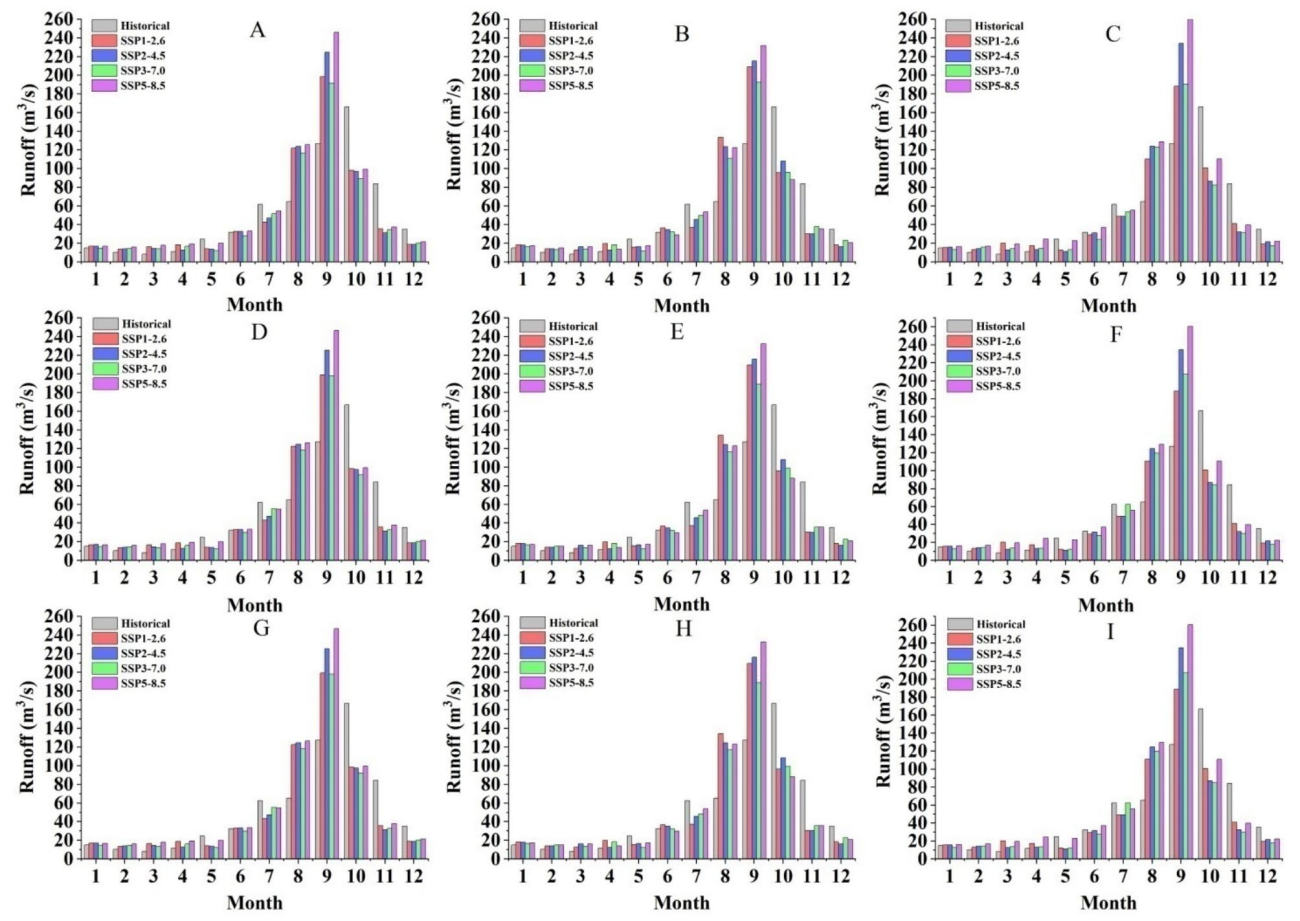
The impact of different land use and climate change combinations on the distribution of intra-annual runoff. (**A**: 2018 land use + 2021–2060 climate scenarios; **B**: 2018 land use + 2021–2040 climate scenarios; **C**: 2018 land use + 2041–2060 climate scenarios; **D**: 2032 land use + 2021–2060 climate scenarios; **E**: 2032 land use  + 2021–2040 climate scenarios; **F**: 2032 land use + 2041–2060 climate scenarios;** G**: 2046 land use + 2021–2060 climate scenarios; **H**: 2046 land use + 2021–2040 climate scenarios; **I**: 2046 land use + 2041–2060 climate scenarios).

The combined influence of climate change and land use change has resulted in significant alterations to the intra-annual runoff distribution pattern, characterized by an early peak and a more concentrated distribution during the wet season (May to October) (Fig. 7). Compared to the intra-annual precipitation distribution, the intra-annual variation of runoff exhibited an average one-month lag during the historical period (1999–2018). However, this delay phenomenon is projected to diminish in the future (2021–2060) (Figs. 5 and 7). In the wet season, the runoff constituted 74.38%, 80.91%, 83.29%, 81.55%, and 81.77% of the total annual runoff under historical, SSP1-2.6, SSP2-4.5, SSP3-7.0, and SSP5-8.5 scenarios, respectively. The historical peak runoff is recorded in October, while the projected future peak runoff is anticipated in September, indicating a trend towards earlier occurrences of future peak runoff. The peak runoff of SSP1-2.6, SSP2-4.5, SSP3-7.0, and SSP5-8.5 scenarios in the period 2021–2060 exhibited respective increases of 32.43 m^3^/s, 58.53 m^3^/s, 29.37 m^3^/s, and 79.74 m^3^/s compared to the historical period. In the near future projections, these increases are projected to be 42.82 m^3^/s, 49.20 m^3^/s, 23.64 m^3^/s, and 65.74 m^3^/s respectively; while in the middle future projections they are expected to reach values of 22.04 m^3^/s, 67 0.85 m^3^/s, 35 0.09 m^3^/s, and 93 0.75 m^3^/s respectively.

## Discussion

### Impact of climate change and land use change on future runoff in DRB

The future runoff of the DRB exhibits an overall increasing trend under the combined influence of climate change and land use change, which aligns with the findings of previous studies^56–60^. The change in runoff serves as a comprehensive reflection of anticipated increases in precipitation, rising temperatures, and the fragmentation of land use. The alteration in precipitation directly influences the spatial distribution of runoff and the overall water resources^8,9,19,61,62^. The DRB is located in a tropical region characterized by abundant precipitation, elevated soil moisture content, and runoff generation driven by saturation excess, resulting in a lower threshold for the transition from precipitation to runoff^63,64^. Compared to the average annual precipitation measured during the base period, future precipitation exhibited significant increases. The increase in precipitation is expected to induce significant changes in surface runoff over a relatively short timeframe. The variation in temperature is a significant hallmark of climate change, and its impact on runoff must not be overlooked. The phenomenon of global warming has been corroborated by an abundance of research findings in recent years^65–67^. The increase in temperature will directly augment surface evaporation, while vegetation transpiration is expected to show an initial rise followed by a subsequent decline^12,13,15^. These changes will undoubtedly exert a substantial influence on future runoff dynamics.

Land use change serves as a pivotal indicator of human activities^8,68^. In contrast to the impact of climate change on runoff during the generation stage, land use change primarily influences runoff concentration during subsequent stages^20^. Different land use types exhibit varying infiltration and absorption effects on precipitation, leading to diverse runoff yields^23,48^. The rich biodiversity of forests, along with the interception of precipitation by tree canopies and understory vegetation, enhances their substantial water conservation capacity^69,70^. The water conservation capacity of cultivated land is relatively limited due to significant human disturbance and a homogeneous vegetation cover^71,72^. Within the integrated effects of climate change and land use change on runoff, future land use alterations significantly enhance the generation of runoff. For example, compared with scenario S_0_, in scenario S_1_, climate change contributes to an increase in runoff of 3.31% and 17.78% for SSP5-8.5 in the near and middle future, respectively. While, in scenario S_3_, the combined impacts of climate change and land use change result in an increase in runoff of 3.65% and 18.24% in the near and middle future, respectively. The findings from the land use analysis conducted in 2032 and 2046 reveal a persistent decline in forested areas, accompanied by a continuous expansion of both cultivated and constructed lands (Fig. 6; Table 7). This phenomenon may elucidate the persistent enhancement of DRB runoff associated with land use change. In the DRB region, the impact of land use on runoff is relatively limited. This may be associated with the elevated soil moisture content in tropical regions^64^. Conversely, changes in land use within arid regions constitute the primary factor affecting runoff variations, thereby reinforcing our hypothesis^3,73^. This indicates that runoff alterations display distinct patterns across different precipitation regions, while the impact of regional land use characteristics on runoff changes exhibits considerable variability.

### Attribution analysis of future climate and land use change

Climate change and land use change are highly complex and interconnected evolutionary processes. As global warming intensifies, a range of profound changes is emerging within the atmospheric system^13,15,58–60,74^. Precipitation and temperature variations are critical meteorological variables that can be easily monitored and analyzed, supported by a substantial repository of historical observational data accumulated by humans^3,24,75,76^. Numerous studies have demonstrated that human activities represent a critical factor in driving global warming^13,51,59,60,74,77^. As temperatures rise, surface transpiration intensifies, thereby increasing precipitation; however, the simultaneous occurrence of both precipitation and transpiration contributes to a reduction in temperature. The interplay between water and heat exchange via precipitation and temperature also influences regional geomorphological features, resulting in corresponding changes in land use^23,56,78^.

The uneven distribution of water and heat has led to the delineation of temperature zones globally, resulting in distinct geomorphic features and vegetation cover characteristics^77,79,80^. The melting of glaciers driven by global warming results in rising sea levels, thereby directly affecting the spatial distribution of land and water^81,82^. The transformation of land use is driven by the combined effects of climate and human activities, with anthropogenic interventions introducing greater complexity to these changes. Our study indicates that the trend of runoff is experiencing a significant increase under the influence of future climate conditions, with average increases of 1.43% and 7.72% in runoff projected for the near and middle future, respectively, compared to the base period. Additionally, land use changes are expected to contribute an increase of 0.41% and 1.14% to runoff in the near and middle futures, respectively. Under the combined effects of future climate change and land use changes, runoff is anticipated to rise by 2.99% and 12.55% in the near and middle futures, respectively. This may be associated with a substantial increase in projected future precipitation, along with a decrease in forested areas and the expansion of cultivated and construction land. This is consistent with the results of previous studies^56,83^. Future changes in climate and land use patterns are expected to significantly influence the existing hydrological and thermal balance at the surface, potentially leading to substantial impacts on regional climate and hydrology; thus, it is essential to continue conducting regional hydrological research.

### Model uncertainty analysis

Understanding and analyzing the uncertainties associated with a model can improve the reliability of simulation results. In our research, rigorous criteria are employed for model calibration and validation to improve the precision of simulation results. The uncertainty in global climate model data primarily stems from the projections of future greenhouse gas emission scenarios and the forced responses of climate models to atmospheric emissions^84^. In this study, we utilized EC-Earth3-Veg, which has been empirically validated in a series of investigations as reliable simulation tool for future climate change projections^39–43^. While a single GCM’s projections may exhibit certain advantages in capturing the amplitude of observed multi-decadal precipitation variability, its associated uncertainty could be greater when compared to an ensemble of multiple GCMs^36,85^. Fortunately, the comparison of downscaled historical data from EC-Earth3-Veg for the period 1967–2014 with observed meteorological data from the DRB indicates that both *R*² and *E*_*NS*_ values exceed 0.5, demonstrating a high level of simulation accuracy in the EC-Earth3-Veg dataset, thereby satisfying the criteria for model simulation^51^. While the CA-Markov model predicts future land use changes, it does not fully account for the extent to which future human activities will shape land use^44–46^. To mitigate the uncertain impacts of human activities on future land use within the CA-Markov model, we performed a comparative analysis between the simulated data for 2018 and actual observed data, resulting in a Kappa coefficient of 0.776. This indicates a strong simulation performance and demonstrates adherence to the model’s accuracy requirements.

The SWAT model is a semi-distributed hydrological model that integrates specific physical processes. However, the simulation results may be influenced by uncertainty arising from the stringent accuracy requirements for driving data and the numerous model parameters^86,87^. In response to this, we conducted a sensitivity analysis on the parameters related to runoff simulation in the SWAT model using SWAT-CUP and evaluated their sensitivity through the T-test method. The inevitability of model uncertainty presents a challenge; however, we can utilize systematic verification methods to confine the error within an acceptable range. In the future, given the escalating complexity of environmental changes arising from human-land interactions, it is essential to persist in investigating model uncertainty.

## Conclusion

In this study, we employed the downscaling technique to examine various scenarios of the EC-Earth3-Veg climate model within the framework of CMIP6. Furthermore, we integrate the predictive outcomes of the CA-Markov model for future land use changes to collaboratively drive the SWAT model and assess potential variations in future runoff within the DRB. The key findings are as follows:

1. In the future (2021–2060), precipitation in the DRB is projected to exhibit an increasing trend under scenarios SSP1-2.6, SSP3-7.0, and SSP5-8.5, whereas a declining trend is anticipated in scenario SSP2-4.5. In comparison to the historical period, a decreasing trend in spring and winter precipitation is evident, while an increasing trend is observed in summer and autumn. The temperature across all four climate scenarios demonstrates a statistically significant increasing trend (*P* < 0.05). Specifically, the projected rate of temperature increase for SSP1-2.6, SSP2-4.5, SSP3-7.0, and SSP5-8.5 is estimated to be 0.24 °C/(10 years), 0.36 °C/(10 years), 0.36 °C/(10 years), and 0.50 °C/(10 years) respectively. The monthly temperature variation exhibits consistent patterns across different scenarios.
2. The predominant land uses included artificial forests, natural forests, and cultivated lands, which accounted for 57.64%, 19.92%, and 14.79% respectively. As a result of the progressive intensification of human activities and stringent governmental protection measures for natural forests, the changes in artificial forests, natural forests, and cultivated lands by 2032 were recorded as − 4.45%, − 0.12%, and 4.10% respectively; whereas by 2046, these changes were noted as − 5.39%, − 0.13%, and 4.41% respectively.
3. The comparison of scenarios S_1_, S_2_, and S_3_ indicates that the combined effects of climate change and land use in the DRB significantly amplify runoff generation. While land use change consistently facilitates runoff, its influence remains limited. Climate change emerges as the primary driver of changes in runoff patterns. In the future, the cumulative effects of climate change and land use change on runoff variations will persistently intensify. The temporal distribution of future runoff is shifted forward by one month relative to the historical period, exhibiting a greater degree of concentration during the wet season. In comparison to the historical period, the projected scenarios of SSP1-2.6, SSP2-4.5, SSP3-7.0, and SSP5-8.5 indicate an expected increase in runoff during the wet season of 6.53%, 8.91%, 7.17%, and 7.39% respectively.

The research findings enhance the understanding of hydrological processes related to runoff in tropical deforestation areas under various future climate scenarios and land-use change patterns. These findings can serve as a valuable reference for future flood prevention, drought mitigation, and water resource management in the DRB. Furthermore, they hold significant implications for water resource management in similar basins.

## Electronic supplementary material

Below is the link to the electronic supplementary material.

Supplementary Material 1

## Data Availability

Relevant data during the current study are available from the corresponding author on reasonable request.

## References

[1] Bahddou, S. et al. Changes in soil surface properties under simulated rainfall and the effect of surface roughness on runoff, infiltration and soil loss. *Geoderma* **431**10.1016/J.GEODERMA.2023.116341 (2023).

[2] Cordeiro, M. R. C. et al. Simulating the hydrological impacts of land use conversion from annual crop to perennial forage in the Canadian prairies using the cold regions hydrological modelling platform. *Hydrol. Earth Syst. Sci.* **26**, 5917–5931 (2022).

[3] Fu, J., Liu, B., Wang, W. & Xu, F. E. Evaluating main drivers of runoff changes across China from 1956 to 2000 by using different budyko-based elasticity methods. *J. Environ. Manage.* **329**, 117070 (2023). 36549061 10.1016/j.jenvman.2022.117070

[4] Guo, W. et al. Quantitative evaluation of runoff variation and its driving forces based on multi-scale separation framework. *J. Hydrology: Reg. Stud.* **43**10.1016/J.EJRH.2022.101183 (2022).

[5] Adam, D. World population hits eight billion—Here’s how researchers predict it will grow. *Nature*10.1038/D41586-022-03720-6 (2022). 36380029 10.1038/d41586-022-03720-6

[6] Li, Y. et al. Multi-model analysis of historical runoff changes in the Lancang-Mekong River Basin—Characteristics and uncertainties. *J. Hydrol.* **619**10.1016/J.JHYDROL.2023.129297 (2023).

[7] Zhou, S., Yu, B., Lintner, B. R., Findell, K. L. & Zhang, Y. Projected increase in global runoff dominated by land surface changes. *Nat. Clim. Change*. **13**, 442–449. 10.1038/s41558-023-01659-8 (2023).

[8] Lucila, C., Karim, T., Gonzalo, O. & Manuel, G. Climate and land use changes on streamflow and subsurface recharge in the Fluvià Basin, Spain. *Water* **8**, 228 (2016).

[9] Singh, D. et al. Machine-learning- and deep-learning-based streamflow prediction in a hilly catchment for future scenarios using CMIP6 GCM data. *Hydrol. Earth Syst. Sci.* **27**, 1047–1075 (2023).

[10] Christopher, J. K. & Shawn, P. S. Impacts of recent climate change on Wisconsin corn and soybean yield trends. *Environ. Res. Lett.* **3**, 034003 (2008).

[11] Zhang, G. et al. Quantifying the impacts of agricultural management practices on the water use efficiency for sustainable production in the Loess Plateau region: a meta-analysis. *Field Crops Res.* **291**10.1016/J.FCR.2022.108787 (2023).

[12] Asseng, S. et al. Rising temperatures reduce global wheat production. *Nat. Clim. Change*. **5**, 143–147 (2015).

[13] Batke, S. P., Yiotis, C., Elliott-Kingston, C., Holohan, A. & McElwain, J. Plant responses to decadal scale increments in atmospheric CO_2_ concentration: Comparing two stomatal conductance sampling methods. *Planta: Int. J. Plant. Biology*. **251**, 52 (2020). 10.1007/s00425-020-03343-z PMC6965045 31950281

[14] Leon, H. A., Vijaya, G. K., Joseph, C. V. V. & Kenneth, J. B. Elevated CO_2_ increases water use efficiency by sustaining photosynthesis of water-limited maize and sorghum. *J. Plant Physiol.* **168**, 1909–1918 (2011). 21676489 10.1016/j.jplph.2011.05.005

[15] Bunce, J. A. Effects of pulses of elevated carbon dioxide concentration on stomatal conductance and photosynthesis in wheat and rice. *Physiol. Plant.* **149**, 214–221 (2013). 23368841 10.1111/ppl.12026

[16] Shrestha, S., Bhatta, B., Shrestha, M. & Shrestha, P. K. Integrated assessment of the climate and landuse change impact on hydrology and water quality in the Songkhram River Basin, Thailand. *Sci. Total Environ.* **643**, 1610–1622. 10.1016/j.scitotenv.2018.06.306 (2018). 30189577 10.1016/j.scitotenv.2018.06.306

[17] Foley, J. A. et al. Solutions for a cultivated planet. *Nature* **478**, 337–342 (2011). 21993620 10.1038/nature10452

[18] Siebert, S. et al. Groundwater use for irrigation—A global inventory. *Hydrol. Earth Syst. Sci.* **14**, 1863–1880. 10.5194/hess-14-1863-2010 (2010).

[19] Tian, X. et al. Climate change impacts on regional agricultural irrigation water use in semi-arid environments. *Agric. Water Manage.* **281**10.1016/j.agwat.2023.108239 (2023).

[20] Song, J., Yang, Z., Xia, J. & Cheng, D. The impact of mining-related human activities on runoff in northern Shaanxi, China. *J. Hydrol.* **598**10.1016/J.JHYDROL.2021.126235 (2021).

[21] Yu, Y. et al. Natural revegetation has dominated annual runoff reduction since the grain for green program began in the Jing River Basin, Northwest China. *J. Hydrol.* **625**10.1016/j.jhydrol.2023.129978 (2023).

[22] Alonso, J., Silveira, L. & Vervoort, R. W. Assessing effects of afforestation on streamflow in Uruguay: From small to large basins. *Hydrol. Process.* **38**10.1002/hyp.15272 (2024).

[23] Zuo, Y., Chen, J., Lin, S. & He, K. The runoff changes are controlled by combined effects of multiple regional environmental factors in the alpine hilly region of Northwest China. *Sci. Total Environ.* **862**, 160835. 10.1016/J.SCITOTENV.2022.160835 (2023). 36502985 10.1016/j.scitotenv.2022.160835

[24] Fan, M. et al. Temperature contributes more than precipitation to runoff in the high mountains of Northwest China. *Remote Sens.* **14**, 4015 (2022).

[25] Wang, M., Zhang, Y., Lu, Y., Gao, L. & Wang, L. Attribution analysis of streamflow changes based on large-scale hydrological modeling with uncertainties. *Water Resour. Manage*. **37**, 713–730 (2022).

[26] Chen, H. et al. Quantitative assessment of impact of climate change and human activities on streamflow changes using an improved three-parameter monthly water balance model. *Remote Sens.* **14**, 4411 (2022).

[27] Sofi, M. S. et al. Modeling the hydrological response of a snow-fed river in the Kashmir Himalayas through SWAT and Artificial neural network. *Int. J. Environ. Sci. Technol.* **21**, 3115–3128. 10.1007/s13762-023-05170-7 (2024).

[28] Rautela, K. S., Kumar, D., Gandhi, B. G. R., Kumar, A. & Dubey, A. K. Long-term hydrological simulation for the estimation of snowmelt contribution of Alaknanda River Basin, Uttarakhand using SWAT. *J. Water Supply: Res. Technology-Aqua*. **72**, 139–159. 10.2166/aqua.2023.176 (2023).

[29] Rautela, K. S., Kuniyal, J. C., Goyal, M. K., Kanwar, N. & Bhoj, A. S. Assessment and modelling of hydro-sedimentological flows of the eastern river Dhauliganga, north-western Himalaya, India. *Nat. Hazards*. **120**, 5385–5409. 10.1007/s11069-024-06413-7 (2024).

[30] Rautela, K. S., Gupta, V., Devi, J. P., Majeed, L. R. & Kuniyal, J. C. Modeling stage-discharge and sediment-discharge relationships in data-scarce Himalayan River Basin Dhauliganga, Central Himalaya, using neural networks. *CLEAN-SOIL AIR WATER*. 10.1002/clen.202300388 (2024).

[31] Ni, X. et al. Simple additive simulation overestimates real influence: Altered nitrogen and rainfall modulate the effect of warming on soil carbon fluxes. *Glob. Change Biol.* **23**, 3371–3381 (2017). 10.1111/gcb.13588 27935178

[32] Li, J. et al. Hydrological and erosion responses of steep spoil heaps to taproot and fibrous root grasses under simulated rainfalls. *J. Hydrol.* **618**10.1016/J.JHYDROL.2023.129169 (2023).

[33] Feng, Z. et al. Responses of soil greenhouse gas emissions to land use conversion and reversion-A global meta-analysis. *Glob. Change Biol.* **28**, 6665–6678 (2022). 10.1111/gcb.16370 35989422

[34] Li, D., Zhu, L., Xu, W. & Ye, C. Quantifying the impact of climate change and human activities on runoff at a tropical watershed in South China. *Front. Environ. Sci.*10.3389/FENVS.2022.1023188 (2022).

[35] Huang, D. Q., Zhu, J., Zhang, Y. C. & Huang, A. N. Uncertainties on the simulated summer precipitation over Eastern China from the CMIP5 models. *J. Geophys. Research: Atmos.* **118**, 9035–9047. 10.1002/jgrd.50695 (2013).

[36] Chen, L. & Frauenfeld, O. W. A comprehensive evaluation of precipitation simulations over China based on CMIP5 multimodel ensemble projections. *J. Geophys. Research: Atmos.* **119**, 5767–5786. 10.1002/2013jd021190 (2014).

[37] Vandana, K., Islam, A., Sarthi, P. P., Sikka, A. K. & Kapil, H. Assessment of potential impact of climate change on streamflow: A case study of the Brahmani River basin, India. *J. WATER Clim. CHANGE*. **10**, 624–641. 10.2166/wcc.2018.129 (2019).

[38] Abeysingha, N. S., Islam, A. & Singh, M. Assessment of climate change impact on flow regimes over the Gomti River basin under IPCC AR5 climate change scenarios. *J. WATER Clim. CHANGE*. **11**, 303–326. 10.2166/wcc.2018.039 (2020).

[39] Abbas, A. et al. Evaluation and projection of precipitation in Pakistan using the coupled model intercomparison project phase 6 model simulations. *Int. J. Climatol.* **42**, 6665–6684. 10.1002/joc.7602 (2022).

[40] Huang, W. R., Chang, Y. H., Deng, L. & Liu, P. Y. Simulation and projection of summer convective afternoon rainfall activities over Southeast Asia in CMIP6 models. *J. Clim.* **34**, 5001–5016. 10.1175/JCLI-D-20-0788.1 (2021).

[41] Kushwaha, P., Pandey, V. K., Kumar, P. & Sardana, D. CMIP6 model evaluation for mean and extreme precipitation over India. *Pure. appl. Geophys.* **181**, 655–678. 10.1007/s00024-023-03409-5 (2024).

[42] Reddy, N. M. & Saravanan, S. Extreme precipitation indices over India using CMIP6: A special emphasis on the SSP585 scenario. *Environ. Sci. Pollut. Res.* **30**, 47119–47143. 10.1007/s11356-023-25649-7 (2023). 10.1007/s11356-023-25649-7 36732454

[43] Wyser, K., Kjellstrom, E., Koenigk, T., Martins, H. & Doscher, R. Warmer climate projections in EC-Earth3-Veg: The role of changes in the greenhouse gas concentrations from CMIP5 to CMIP6. *Environ. Res. Lett.* **15**10.1088/1748-9326/ab81c2 (2020).

[44] Mostafa, E., Li, X. & Sadek, M. Urbanization trends Analysis using hybrid modeling of fuzzy analytical hierarchical process-cellular Automata-Markov Chain and investigating its impact on land surface temperature over Gharbia City, Egypt. *Remote Sens.* **15**, 843 (2023).

[45] Hou, G., Zhang, H., Liu, Z., Chen, Z. & Cao, Y. Historical reconstruction of aquatic vegetation of typical lakes in Northeast China based on an improved CA-Markov model. *Front. Ecol. Evol.*10.3389/FEVO.2022.1031678 (2022).

[46] Hao, L., He, S., Zhou, J., Zhao, Q. & Lu, X. Prediction of the landscape pattern of the Yancheng Coastal Wetland, China, based on XGBoost and the MCE-CA-Markov model. *Ecol. Ind.* **145**10.1016/J.ECOLIND.2022.109735 (2022).

[47] Zhang, Z. et al. Research on the optimal allocation of agricultural water and soil resources in the Heihe River Basin based on SWAT and intelligent optimization. *Agric. Water Manage.* **279**10.1016/J.AGWAT.2023.108177 (2023).

[48] Wang, Z. et al. A generalized reservoir module for SWAT applications in watersheds regulated by reservoirs. *J. Hydrol.* **616**10.1016/J.JHYDROL.2022.128770 (2023).

[49] Dash, S. S., Sahoo, B. & Raghuwanshi, N. S. SWAT model calibration approaches in an integrated paddy-dominated catchment-command. *Agric. Water Manage.* **278**10.1016/J.AGWAT.2023.108138 (2023).

[50] Sun, Q., Miao, C. & Duan, Q. Extreme climate events and agricultural climate indices in China: CMIP5 model evaluation and projections. *Int. J. Climatol.* **36**, 43–61. 10.1002/joc.4328 (2016).

[51] Moriasi, D. N. et al. Model evaluation guidelines for systematic quantification of accuracy in watershed simulations. *Trans. ASABE*. **50**, 885–900 (2007).

[52] Kapil, H., Sikka, A. K., Sarthi, P. P., Islam, A. & Vandana, K. Assessment of potential impact of climate change on streamflow: A case study of the Brahmani River basin, India. *J. Water Clim. Change*. **10**, 624–641. 10.2166/wcc.2018.129 (2019).

[53] Desai, S., Singh, D. K., Islam, A. & Sarangi, A. Multi-site calibration of hydrological model and assessment of water balance in a semi-arid river basin of India. *Quatern. Int.* **571**, 136–149. 10.1016/j.quaint.2020.11.032 (2021).

[54] Bedewi, S. A. & Arigaw, A. K. Multi-site calibration of hydrological model and the response of water balance components to land use land cover change in a rift valley Lake Basin in Ethiopia. *Sci. Afr.* **15** (2022).

[55] Abbaspour, K. C. et al. A continental-scale hydrology and water quality model for Europe: Calibration and uncertainty of a high-resolution large-scale SWAT model. *J. Hydrol.* **524**, 733–752 (2015).

[56] Zhou et al. Projection of China’s future runoff based on the CMIP6 mid-high warming scenarios. *Scientia Sinica(Terrae)*. **53**, 505–524 (2023).

[57] Zhang, Y. et al. Future global streamflow declines are probably more severe than previously estimated. *Nat. Water*. **1**, 261–271. 10.1038/s44221-023-00030-7 (2023).

[58] Do, H. X. et al. Historical and future changes in global flood magnitude—evidence from a model–observation investigation. *Hydrol. Earth Syst. Sci.* **24**, 1543–1564 (2020).

[59] Cook, B. I. et al. Twenty-First Century Drought projections in the CMIP6 forcing scenarios. *Earth’s Future*. **8**10.1029/2019ef001461 (2020).

[60] Betts, R. A. et al. Projected increase in continental runoff due to plant responses to increasing carbon dioxide. *Nature* **448**, 1037–1041 (2007). 17728755 10.1038/nature06045

[61] Mehdi, B., Ludwig, R. & Lehner, B. Evaluating the impacts of climate change and crop land use change on streamflow, nitrates and phosphorus: A modeling study in Bavaria. *J. Hydrology: Reg. Stud.* **4**, 60–90 (2015).

[62] Li, X., Tian, Y., Sun, J., Wei, Y. & Li, F. Evolutionary effect separation of watershed characteristics for the multi-source contributions to runoff changes in the Yellow River, China. *Ecol. Ind.* **143**10.1016/J.ECOLIND.2022.109398 (2022).

[63] Mavimbela, S. S. W., Dlamini, P. & Rensburg, L. D. Infiltration-excess runoff properties of dryland floodplain soil types under simulated rainfall conditions. *Arid Land. Res. Manage.* **33**, 235–254 (2019).

[64] Huang, P., Li, Z., Yao, C., Li, Q. & Yan, M. Spatial combination modeling framework of saturation-excess and infiltration-excess runoff for semihumid watersheds. *Advances in Meteorology* 1–15 (2016).

[65] Savin, C. et al. Climate processes and drivers in the Pacific and global warming: A review for informing Pacific planning agencies. *Clim. Change*. **176**10.1007/S10584-022-03467-Z (2023).

[66] Nico, W. et al. Global warming overshoots increase risks of climate tipping cascades in a network model. *Nat. Clim. Change*. **13**, 75–82 (2022).

[67] Feng, Y., Romps, I. N. R., Chambers, J. Q. & D. M. & Amazon windthrow disturbances are likely to increase with storm frequency under global warming. *Nat. Commun.* **14**, 101 (2023). 36609508 10.1038/s41467-022-35570-1 PMC9822931

[68] Alamdari, N., Claggett, P., Sample, D. J., Easton, Z. M. & Nayeb, Y. M. Evaluating the joint effects of climate and land use change on runoff and pollutant loading in a rapidly developing watershed. *J. Clean. Prod.* **330**10.1016/J.JCLEPRO.2021.129953 (2022).

[69] Huang, X., Liu, J., Peng, S. & Huang, B. The impact of multi-scenario land use change on the water conservation in central Yunnan urban agglomeration, China. *Ecol. Ind.* **147**10.1016/J.ECOLIND.2023.109922 (2023).

[70] Sun, Z. et al. A healthier water use strategy in primitive forests contributes to stronger water conservation capabilities compared with secondary forests. *Sci. Total Environ.* **851**, 158290. 10.1016/J.SCITOTENV.2022.158290 (2022). 36030869 10.1016/j.scitotenv.2022.158290

[71] Deuschle, D., Minella, J. P. G., Hörbe, T. A. N., Londero, A. L. & Schneider, F. J. A. Erosion and hydrological response in no-tillage subjected to crop rotation intensification in southern Brazil. *Geoderma* **340**, 157–163 (2019).

[72] Quijano, L., Beguería, S., Gaspar, L. & Navas, A. Estimating erosion rates using 137 cs measurements and WATEM/SEDEM in a Mediterranean cultivated field. *Catena* **138**, 38–51 (2016).

[73] He, Y., Yang, H., Liu, Z. & Yang, W. A framework for attributing runoff changes based on a monthly water balance model: An assessment across China. *J. Hydrol.* **615**10.1016/J.JHYDROL.2022.128606 (2022).

[74] Das, P. et al. Historical and projected changes in Extreme High temperature events over East Africa and associated with meteorological conditions using CMIP6 models. *Glob. Planet Change*. **222**10.1016/J.GLOPLACHA.2023.104068 (2023).

[75] Hamed, M. M. et al. Future Köppen-Geiger climate zones over Southeast Asia using CMIP6 Multimodel Ensemble. *Atmos. Res.* **283**10.1016/J.ATMOSRES.2022.106560 (2023).

[76] He, J., Brogniez, H. & Picon, L. Evaluation of tropical water vapour from CMIP6 global climate models using the ESA CCI Water Vapour climate data records. *Atmos. Chem. Phys.* **22**, 12591–12606 (2022).

[77] Gao, X. et al. Changes in global vegetation distribution and Carbon fluxes in response to global warming: Simulated results from IAP-DGVM in CAS-ESM2. *Adv. Atmos. Sci.* **39**, 1285–1307 (2022).

[78] Yang, Y. & Tang, J. Downscaling and uncertainty analysis of future concurrent long-duration dry and hot events in China. *Clim. Change*. **176**10.1007/S10584-023-03481-9 (2023).

[79] I., H. S. P. P. C. & Climate CO_2_ controls on global vegetation distribution at the last glacial maximum: Analysis based on palaeovegetation data, biome modelling and palaeoclimate simulations. *Glob. Change Biol.* **9**, 983–1004 (2003).

[80] Li, H., Renssen, H. & Roche, D. M. Global vegetation distribution driving factors in two dynamic global vegetation models of contrasting complexities. *Glob. Planet Change*. **180**, 51–65 (2019).

[81] Flora, G. Daily briefing: Melting Himalayan glaciers will affect more than one billion people. *Nature*10.1038/D41586-022-03230-5 (2022). 10.1038/d41586-022-03230-5 36216950

[82] Young, J. C. et al. A changing hydrological regime: Trends in magnitude and timing of glacier ice melt and glacier runoff in a high latitude coastal watershed. *Water Resour. Res.* **57**10.1029/2020WR027404 (2021).

[83] Xing, W., Wang, W., Zou, S. & Deng, C. Projection of future runoff change using climate elasticity method derived from Budyko framework in major basins across China. *Glob. Planet Change*. **162**, 120–135 (2018).

[84] Perraud, J. M., Wang, B., Chiew, F. H. S., Vaze, J. & Teng, J. Estimating the relative uncertainties sourced from GCMs and hydrological models in modeling climate change impact on runoff. *J. Hydrometeorol.* **13**, 122–139. 10.1175/jhm-d-11-058.1 (2012).

[85] Islam, A., Ahuja, L. R., Garcia, L. A., Ma, L. & Saseendran, A. S. Modeling the effect of elevated CO_2_ and climate change on reference evapotranspiration in the semi-arid central great plains. *Trans. ASABE*. **55**, 2135–2146 (2012).

[86] Chordia, J., Panikkar, U. R., Srivastav, R. & Shaik, R. U. Uncertainties in prediction of streamflows using SWAT model—role of remote sensing and precipitation sources. *Remote Sens.* **14**, 5385 (2022).

[87] Karlsson, I. B. et al. Combined effects of climate models, hydrological model structures and land use scenarios on hydrological impacts of climate change. *J. Hydrol.* **535**, 301–317. 10.1016/j.jhydrol.2016.01.069 (2016).

