# Supplementary material for: Effects of future climate and land use changes on runoff in tropical regions of China
Source: Sci Rep. 2024 Dec 28;14:30922. doi: 10.1038/s41598-024-81754-8 (PMC11681258; doi:10.1038/s41598-024-81754-8)
Supplement: Supplementary file 1 — Supplementary Material 1 [file 41598_2024_81754_MOESM1_ESM.docx]

**Effects of future climate and land use changes on runoff in tropical regions of China**

Shiyu Xue^1＃^, Xiaohui Guo^1＃^, Yanhu He^2^, Hao Cai^1^, Jun Li^1^, Lirong Zhu^3^, Changqing Ye^1,^^4*✉^

^*✉^corresponding author: Changqing Ye

**^＃^**These two authors contributed equally to this work.

[1] School of Ecology, Hainan University, Haikou, 570228, China.

[2] Institute of Environmental and Ecological Engineering, Guangdong University of Technology, Guangzhou, 510006, China.

[3] School of Tourism, Hainan University, Haikou, 570228, China.

[4] Key Laboratory of Agro-Forestry Environmental Processes and Ecological Regulation of Hainan Province, Haikou, 570228, China.

**Address for correspondence:** Dr. Changqing Ye, School of Ecology, Hainan University, Haikou, China. Tel: +86 13637653039, e-mail: [yechangqing2001@hotmail.com](mailto:yechangqing2001@hotmail.com)


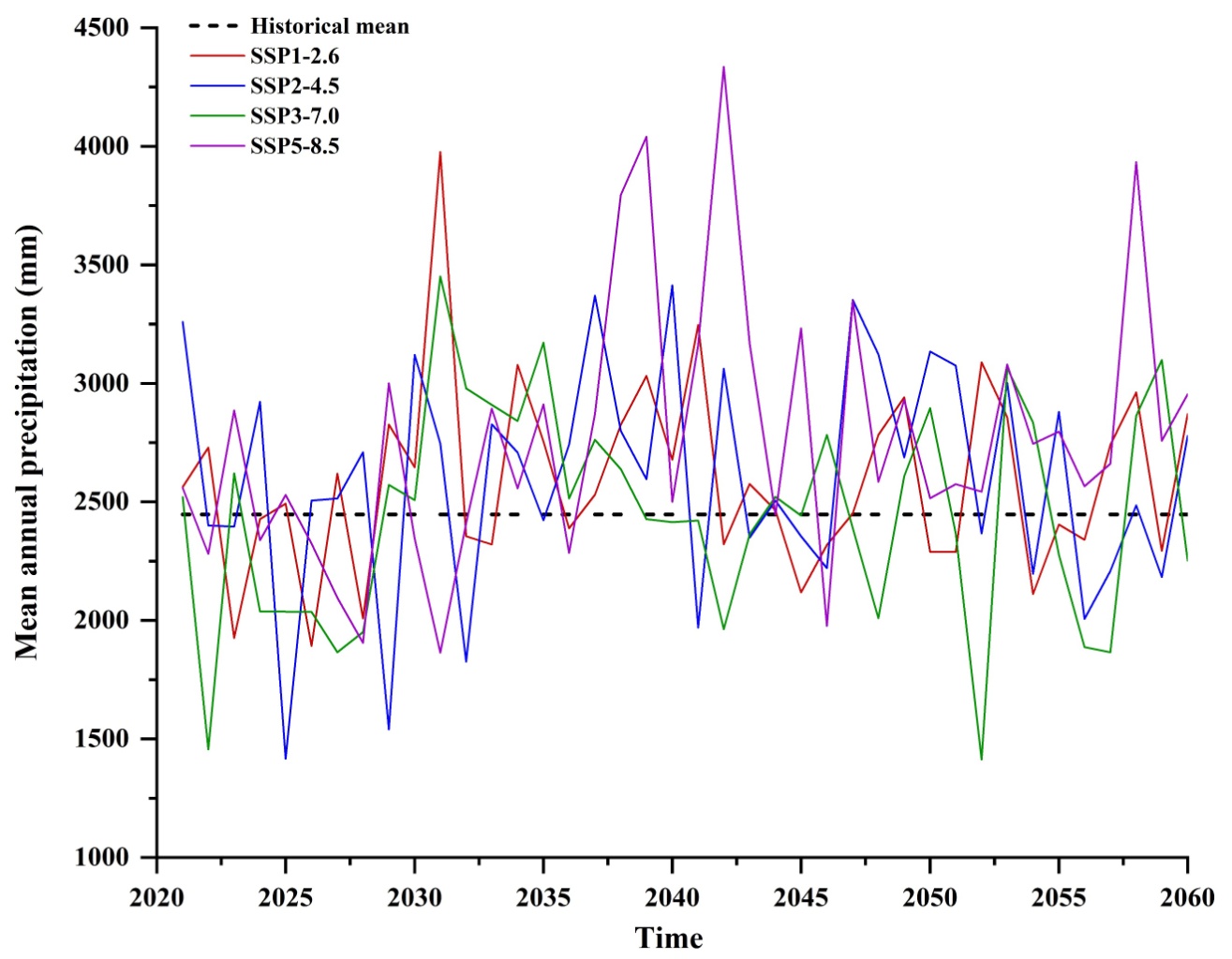


**Fig.S1** Trends in precipitation variability under different climate change scenarios


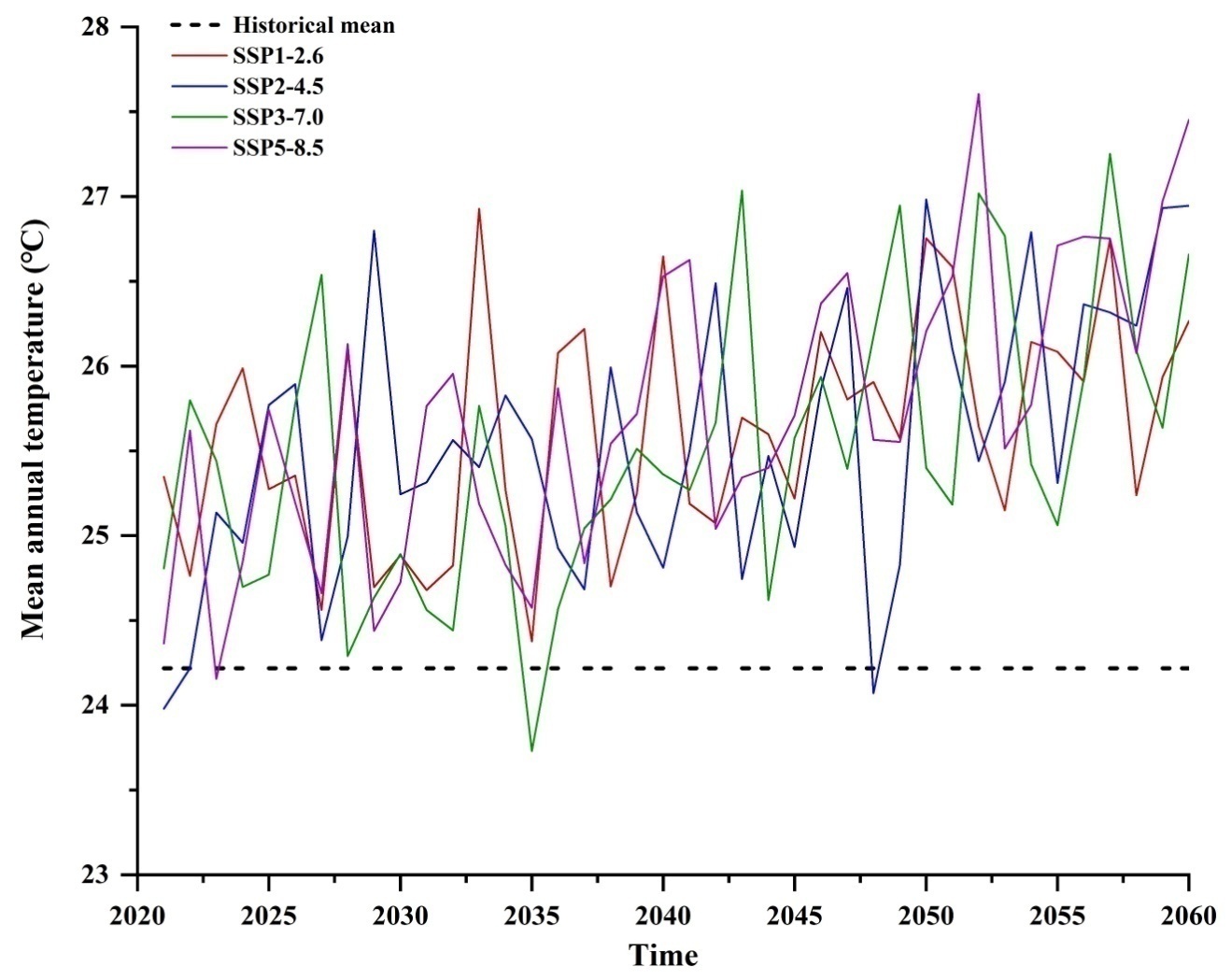


**Fig.S2** Trends of future annual average temperature changes under different climate scenarios


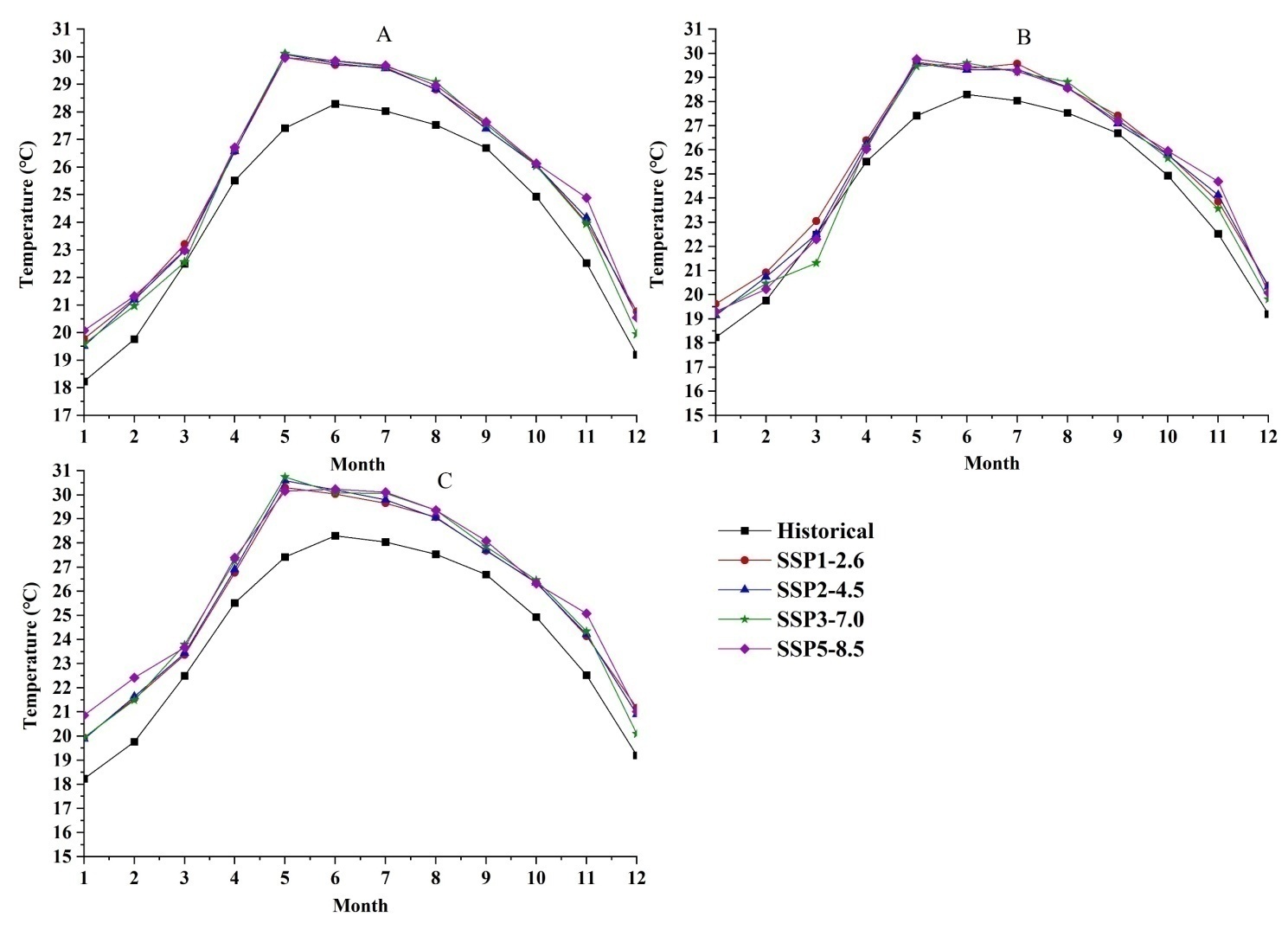


**Fig.S3** Temporal variations in monthly temperature patterns across different climate scenarios (A:2021-2060; B:2021-2040; C:2041-2060)
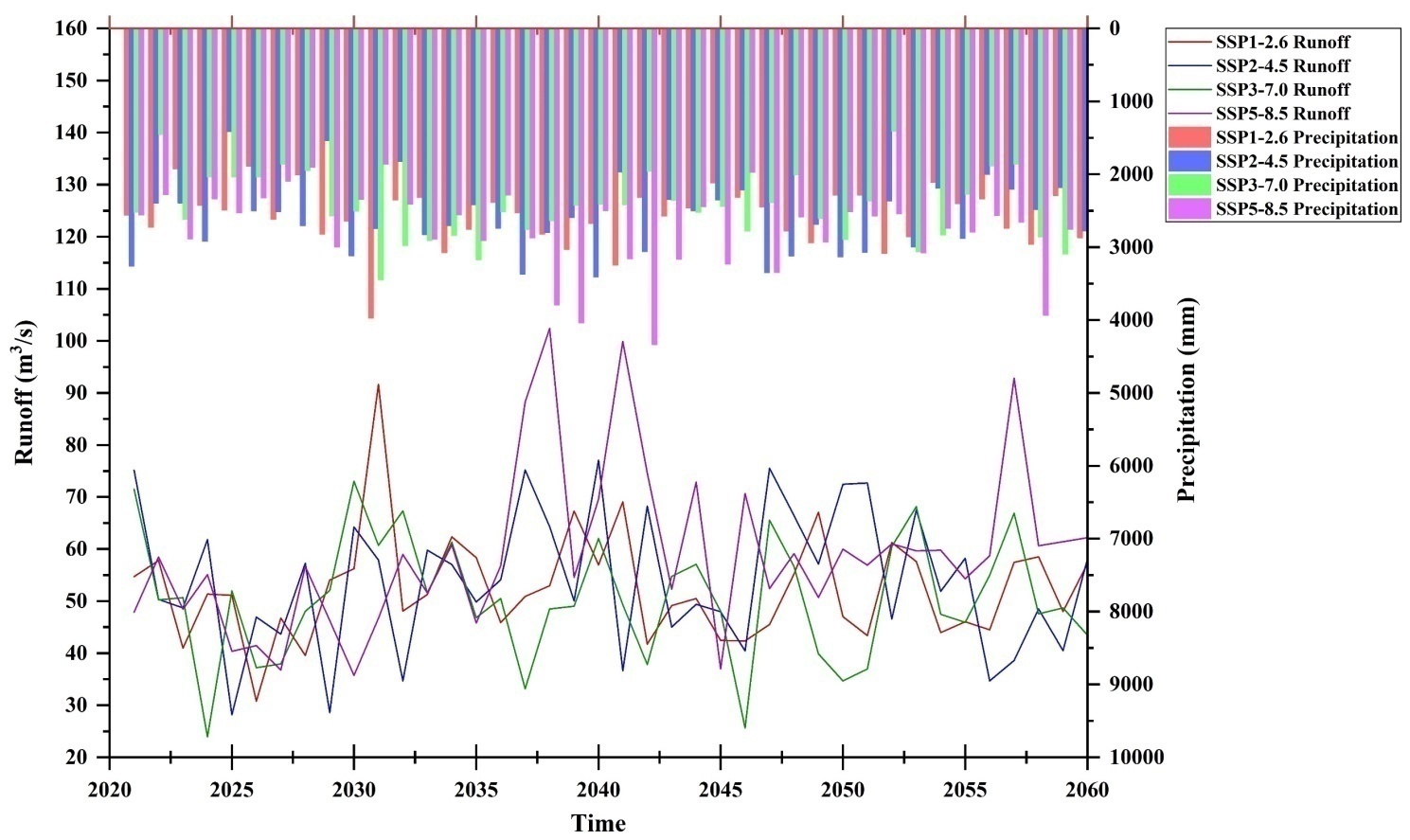


**Fig.S4** Impact of climate change on runoff


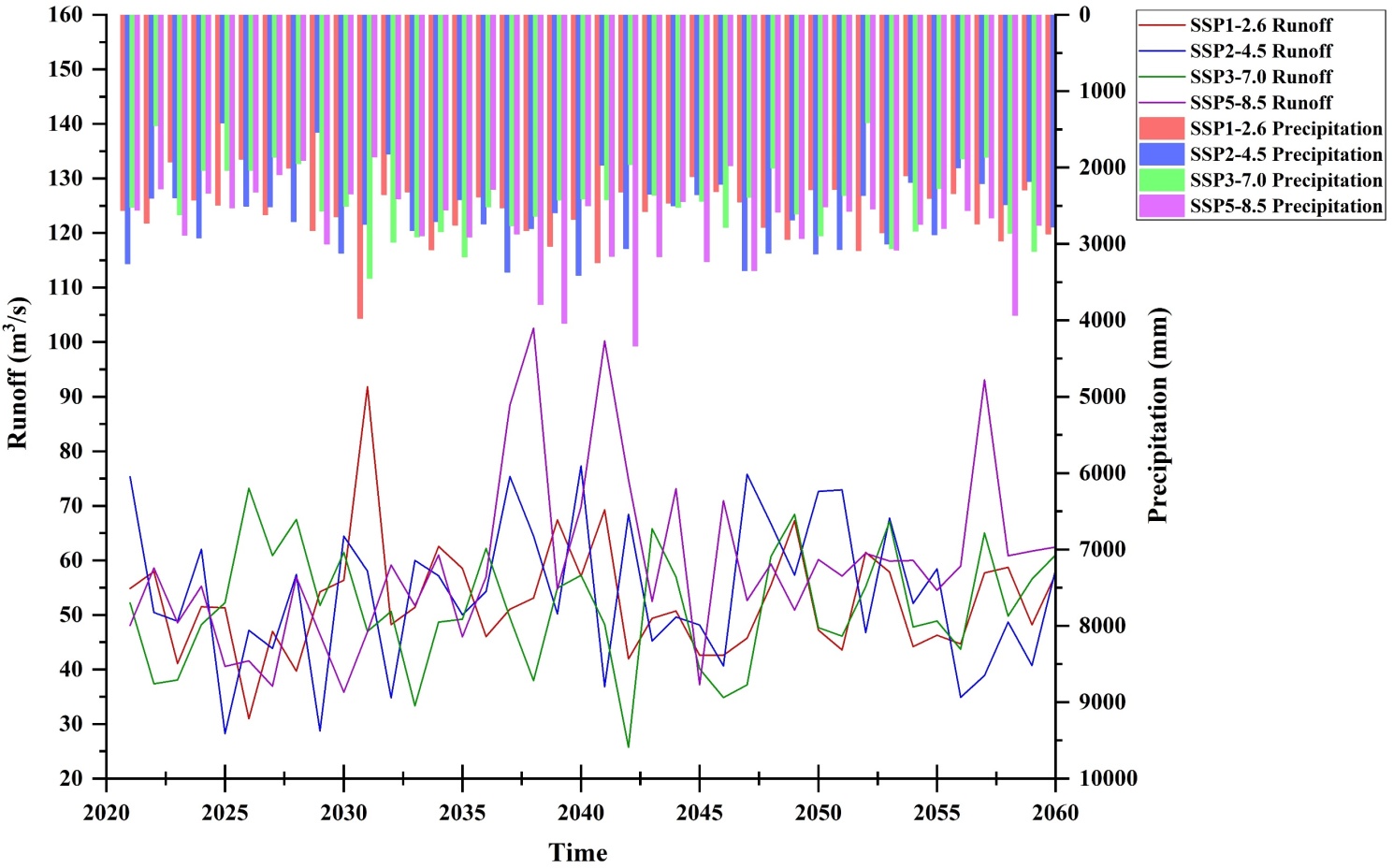


**Fig.S5** The inter-annual variation of runoff under the combined impact of climate change and land use change
